# The current state of complex systems research on socioeconomic inequalities in health and health behavior—a systematic scoping review

**DOI:** 10.1186/s12966-024-01562-1

**Published:** 2024-02-05

**Authors:** Andrea L. Mudd, Michèlle Bal, Sanne E. Verra, Maartje P. Poelman, John de Wit, Carlijn B. M. Kamphuis

**Affiliations:** 1 Department of Interdisciplinary Social Science- Public Health, Utrecht University, PO Box 80140, 3508 TC Utrecht, The Netherlands; 2 Chair Group Consumption and Healthy Lifestyles, Wageningen University & Research, Hollandseweg 1, 6706 KN Wageningen, the Netherlands

**Keywords:** Complex systems, Socioeconomic inequalities, Health and well-being, Health behavior, Systematic scoping review, Causal loop diagram

## Abstract

**Background:**

Interest in applying a complex systems approach to understanding socioeconomic inequalities in health is growing, but an overview of existing research on this topic is lacking. In this systematic scoping review, we summarize the current state of the literature, identify shared drivers of multiple health and health behavior outcomes, and highlight areas ripe for future research.

**Methods:**

SCOPUS, Web of Science, and PubMed databases were searched in April 2023 for peer-reviewed, English-language studies in high-income OECD countries containing a conceptual systems model or simulation model of socioeconomic inequalities in health or health behavior in the adult general population. Two independent reviewers screened abstracts and full texts. Data on study aim, type of model, all model elements, and all relationships were extracted. Model elements were categorized based on the Commission on Social Determinants of Health framework, and relationships between grouped elements were visualized in a summary conceptual systems map.

**Results:**

A total of 42 publications were included; 18 only contained a simulation model, 20 only contained a conceptual model, and 4 contained both types of models. General health outcomes (e.g., health status, well-being) were modeled more often than specific outcomes like obesity. Dietary behavior and physical activity were by far the most commonly modeled health behaviors. Intermediary determinants of health (e.g., material circumstances, social cohesion) were included in nearly all models, whereas structural determinants (e.g., policies, societal values) were included in about a third of models. Using the summary conceptual systems map, we identified 15 shared drivers of socioeconomic inequalities in multiple health and health behavior outcomes.

**Conclusions:**

The interconnectedness of socioeconomic position, multiple health and health behavior outcomes, and determinants of socioeconomic inequalities in health is clear from this review. Factors central to the complex system as it is currently understood in the literature (e.g., financial strain) may be both efficient and effective policy levers, and factors less well represented in the literature (e.g., sleep, structural determinants) may warrant more research. Our systematic, comprehensive synthesis of the literature may serve as a basis for, among other things, a complex systems framework for socioeconomic inequalities in health.

**Supplementary Information:**

The online version contains supplementary material available at 10.1186/s12966-024-01562-1.

## Background

Socioeconomic inequalities in health remain a pressing concern. Despite many years of research and policies aimed at reducing these inequalities, those who are best off in society continue to live longer and healthier lives than those who are worse off, and evidence suggests this socioeconomic gradient is widening in high-income countries [1–3]. There is increasing consensus that understanding how socioeconomic inequalities are formed and maintained requires considering the functioning of the complex system in its entirety, as traditional approaches focused on causal effects of single factors have yielded unsatisfying explanations [4, 5].

Complex systems are characterized by *heterogeneous system elements*, which are the entities within the system (e.g., people, resources), at various *levels of influence*, such as individual- and structural-level determinants of health [6]. System elements are *related to each other* within and across levels of influence, and some of these relationships form *feedback loops*, which are sets of relationships that reinforce or balance each other out over time [7]. The system *adapts* to internal and external changes introduced to the system in a *non-linear and dynamic* way, meaning that changes to the system can have disproportionate effects that change over time [4, 8]. Complex systems contain *emergent patterns*, such that system-level behavior cannot always be attributed to its individual parts [7, 9].

Conceptual and simulation approaches are two broad types of applications of complex systems. A conceptual approach entails a representation of the causal structure of a complex system, often visualized as a conceptual model or framework. A simulation approach entails a formalization of the causal structure of a complex system, using equations to quantify how model elements relate to each other [4]. Conceptual and simulation approaches are often complementary (conceptual systems models can inform the structure of simulation models) [8, 10], and both approaches can provide valuable insight into the systems they aim to represent.

Interest in applying a complex systems approach to socioeconomic inequalities in health is growing. Some existing reviews have described the application of systems thinking in public health [11], of simulation models in public health [12], or of simulation models to socioeconomic inequalities in health [13, 14]. Others have summarized complex systems approaches for specific outcomes, like diet [15], obesity [16, 17], and food environments [18]. To gauge our current understanding of socioeconomic inequalities in health and health behavior from a complex systems perspective, a comprehensive review is needed that encompasses: both conceptual and simulation approaches, explicit consideration of socioeconomic inequalities, and a broad range of health and health behavior outcomes. Conceptual and simulation approaches are both important and often complement each other, so an in-depth understanding of the content (including mechanisms and how they are interrelated) of existing models using both of these approaches is useful. Explicit consideration of socioeconomic inequalities is crucial, as complex systems approaches that do not take socioeconomic inequalities or socioeconomic position (SEP) into account cannot provide insight into how these inequalities are developed, maintained, and mitigated. Finally, consideration of a broad range of health and health behavior outcomes is of value, as these outcomes are likely interlinked and may be influenced by shared drivers. Indeed, a growing body of research on syndemics and multimorbidity highlights that chronic health issues often compound one another and are interlinked in their influence on overall health and well-being [12, 19, 20].

The purpose of this study is to synthesize existing literature on the dynamics underlying socioeconomic inequalities in health and health behavior modeled from a complex systems perspective. To do this, we conduct a systematic scoping review of published peer-reviewed studies on this subject. We use the term “systematic scoping review” because our review combines the rigor of a systematic literature review with the general purpose of a scoping review, to identify, summarize, and map available evidence on a topic [21]. In our review, we aim to: 1. Summarize key study and model characteristics, including study aims, types of models, measures of SEP, determinants, and model outcomes and 2. Visualize the current state of research in a summary conceptual systems map that allows us to identify shared drivers of multiple outcomes. These insights may help inform future study designs for researchers wishing to apply a complex systems approach to similar topics and could serve as a systematic, literature-based starting point for the development of a complex systems framework for understanding socioeconomic inequalities in health. This manuscript is focused on the *content* of the existing literature. In a separate, forthcoming short report [22], we delve into the complex systems *methods* employed.

## Methods

The protocol for our systematic scoping review was registered with the International Prospective Register of Systematic Reviews (PROSPERO) (registration ID: CRD42021286866). Our review adhered to the Preferred Reporting Items for Systematic Reviews and Meta-Analyses (PRISMA) checklist (see Supplementary File 1).

### Search strategy

The search terms and search strategy were developed in collaboration with an information specialist. SCOPUS, Web of Science, and PubMed databases were searched from database start dates to April 2023 for English-language peer-reviewed studies and conference proceedings. Supplementary File 2 contains the full search strategy. Two independent reviewers (AM was the first reviewer, and CK, MB, SV, and MP were second reviewers) screened titles and abstracts according to predefined eligibility criteria. Discrepancies were discussed between reviewers and, if needed, with the full research team, until agreement was reached. This process was repeated to screen full texts that were included in the title and abstract screening phase.

### Eligibility criteria

The target population was the adult general population in high-income countries belonging to the Organization for Economic Co-operation and Development (OECD). While health inequalities are a prescient issue across the globe, we expected that the main mechanisms underlying health inequalities in different populations and contexts, such as youth or populations of lower-income countries, may be somewhat distinct from the main mechanisms relevant for adults in higher-income countries [23, 24]. Studies were included if they contained a conceptual or simulation model of socioeconomic inequalities in health or health behavior developed from a complex systems perspective. More specifically, models were required to include SEP in general or a specific measure of SEP (i.e., educational level, income, occupation). Models were required to include a health or health behavior outcome relevant for the adult general population (health or well-being in general, chronic diseases like obesity, or associated health behaviors like diet and physical activity). To make the literature search feasible in scope, we searched for studies that self-identified as applying a complex systems perspective [6]. Because we aimed to summarize the content of the complex systems models, we required that the studies present an original or adapted model in the publication.

### Data extraction and analysis

Data on study characteristics and model content were extracted by one reviewer (AM). Two reviewers (SV and MP) validated the data extraction performed by the first reviewer on 20% of the included studies, and any discrepancies were discussed between the reviewers and, if needed, with the full research team, until agreement was reached. Insights from these discussions were applied to the data extracted from all studies included in the review. Study characteristics included the main aim of the study, a description of the model, and whether the relationships reported in the model specified the direction (going to and from certain model elements), polarity (positive or negative), or magnitude (strength) of the relationships. Model content comprised all elements and relationships, including direction and polarity (when specified), contained in each model.

**Fig. 1:**
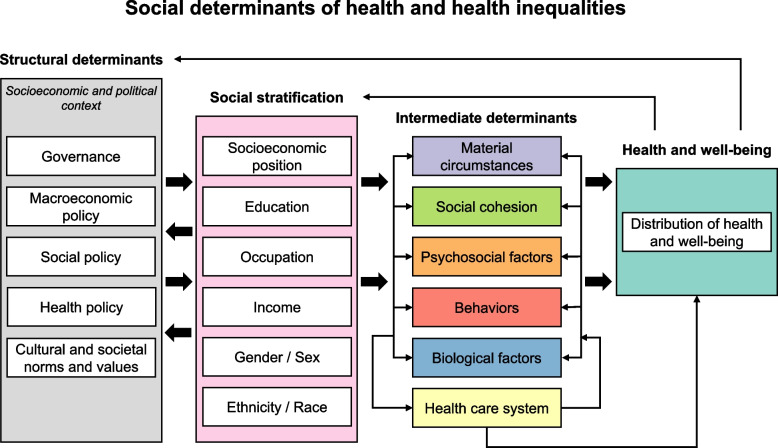
CSDH framework, replicated from CSDH and Qi et al. [25, 26]

As a starting point for analyzing the model content, all elements from the models in the studies included in our review were sorted into the categories in the existing Commission on Social Determinants of Health (CSDH) framework developed by the World Health Organization, which is depicted in Fig. 1 [25–27]. The CSDH framework accounts for interrelations between health behavior, health outcomes, and mechanisms at multiple levels of influence, making it a useful tool for categorizing the content of the models included in the review. The categories we used were: health and well-being, the health care system, material circumstances, behavior, psychosocial factors, biological factors, social cohesion, SEP, gender, ethnicity, governance, macroeconomic policies, social policies, public policies, and culture and societal norms and values. In the event that model elements did not fit into these CSDH framework categories, additional categories were created. Within each CSDH framework-based category, related model elements were grouped together; for example, the grouped element called “stress” included elements from specific models such as “perceived stress”, “mental health stressors”, and other closely related elements. This grouping process was literature-driven, meaning that element groups were only created if multiple related elements were identified in the literature. Whenever possible, the element groups were assigned names that allowed for meaningful interpretation of the polarity of relationships between element groups (e.g., “healthy diet” instead of “diet”). This approach meant that we were sometimes required to apply our interpretation of whether a model element was health-promoting or not. This interpretation was informed by a close reading of the studies and, if needed, we consulted additional literature about specific model elements that our team did not have expertise in. For example, although there is debate about the optimal range of fluoride intake [28], we interpreted a higher fluoride intake as health promoting where the variable ranged from no fluoride intake to fluoride intake within generally acceptable ranges [29]. The categorization process was iterative and collaborative. One reviewer (AM) performed the initial CSDH framework-based categorization and element grouping, which was then discussed and adjusted with two reviewers (CK and MB) until all elements were categorized into element groups.

A matrix system in Microsoft Excel was used to organize all extracted relationships from the models in the studies included in our review. Both axes of the matrix contained all element groups, and each cell in the matrix contained the specific model relationships *from* one element group (vertical axis) *to* another element group (horizontal axis). For example, the matrix cell representing relationships *from* cultural and social norms *to* healthy diet could contain multiple specific relationships between elements belonging to these element groups from multiple studies. In each matrix cell, the relationship polarities and references to the source studies were recorded.

The relationships between element groups were visualized in a summary conceptual systems map, in the form of a causal loop diagram (CLD), which is a representation of the causal structure of a dynamic system that consists of variables (elements), causal links between variables (relationships), and the polarity of the causal links [30]. The CLD was built in the freely available Kumu software [31]. The polarities of the relationships between the element groups were derived from an analysis of the polarities of all relationships in the corresponding matrix cell. If some relationship polarities in a matrix cell were specified and others were unspecified, we assigned the specified polarity to the relationship in the summary conceptual systems map. If some of the specified polarities were positive and some were negative within a matrix cell, the polarity was marked as conflicting. In cases where relationship polarity was stated but we could not meaningfully interpret one of the element groups as health-promoting or health-suppressing (e.g., household demographics, which includes specific elements like marital status and number of children), the polarity was marked as unspecified. The boundaries of the summary conceptual systems map were driven by the literature, meaning that we included all available information from the studies identified in the review. For the purposes of this analysis, a model element in the summary conceptual systems map was considered a direct shared driver of multiple outcomes if it had a direct influence on 2 or more health or health behavior outcomes and was influenced by at least one measure of SEP.

### Quality assessment

The quality assessment was performed by one reviewer (AM). Two reviewers (SV and MP) validated the quality assessment for a total of 20% of the included studies. Any discrepancies were discussed between the reviewers and, if needed, with the full research team, until agreement was reached. Insights from these discussions were applied to the quality assessment for all studies included in the review. Due to the subjective nature of the quality assessment, the main focus was to be consistent in our assessment for all studies. We used two traffic light-based quality assessments to evaluate the evidence each model was based on and how each study applied key concepts of a complex systems approach. In both assessments, green indicated high quality, yellow indicated medium quality, and red indicated low quality. In the absence of a standard quality assessment for complex systems models, we adapted an existing similar traffic light-based assessment [6]. The key concepts of a complex systems approach were selected based on literature describing complex systems approaches and the existing traffic light system [4, 6, 8]. The purpose of the quality assessment of the model evidence base was to evaluate to what extent the authors based the model on evidence, regardless of what the chosen evidence base was. Complex systems models may be based on literature (e.g., [32]), empirical data (e.g., [33]), iterative processes involving stakeholders (e.g., [34]), or a combination of these (e.g., [35]), which are all legitimate types of evidence to be used according to the aim and approach of the study. An overview of the two quality assessments, including definitions of the key concepts of complex adaptive systems, can be found in Supplementary File 3.

## Results

**Fig. 2:**
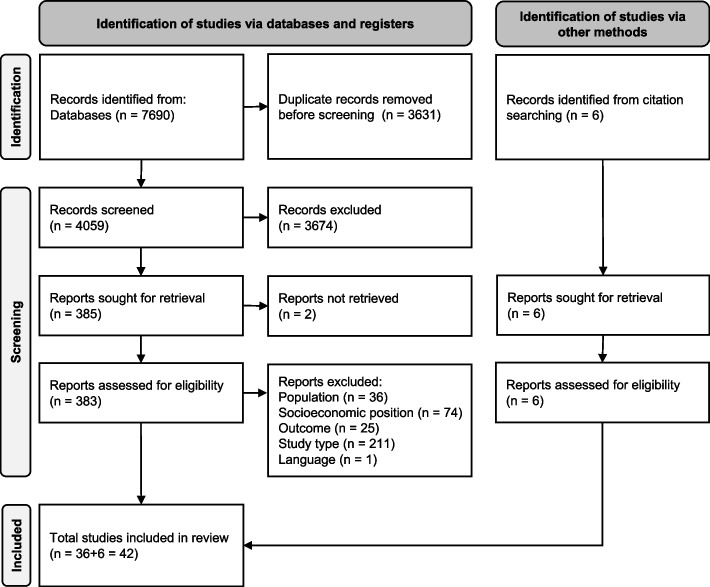
PRISMA flow chart

After removing duplicates, 4059 abstracts were screened. Of these, 383 full texts were screened and 36 were included according to the eligibility criteria. An additional 6 studies were identified by screening citations via hand searching, resulting in a total of 42 studies published between 1987 and 2023 included in the review. The PRISMA flow chart details the identification, screening, and inclusion decisions made (Fig. 2). In the full text screening phase, the most common reason for exclusion was study type, meaning that studies did not report applying a complex systems approach to develop a conceptual or simulation model.

### Key study and model characteristics

In the 42 included studies, 4 contained both a conceptual and simulation model [29, 33, 36, 37], 18 only contained a simulation model [34, 38–54], and 20 only contained a conceptual systems model [32, 35, 55–72]. While all studies included at least one measure of SEP and stated, broadly, that a complex systems approach was applied, about a third of the included studies mentioned socioeconomic inequalities (*N* = 12) or a complex systems approach (*N* = 15) in their study aim.

Table 1 shows an overview of key study and model characteristics. Types of simulation models included agent-based models (*N* = 15) [29, 34, 36–40, 46–53], system dynamics models (*N* = 6) [33, 41, 42, 44, 45, 54], and a dynamic microsimulation (*N* = 1) [43]. The types of conceptual systems models were more varied, and CLD (*N* = 7) [35, 59, 67–69, 71, 72] was the most common.

**Table 1:** Key study and model characteristics of studies included in the review

First author last name	Publication year	Type of model	Model evidence base	Measure(s) of SEP	Modelled health behaviors	Modelled health outcomes
*Studies containing a conceptual model or framework*						
Ansari [55]	2003	Eco-epidemiological theoretical framework	Literature and author expertise (not explicitly stated)	SEP (in general)	Health behavior in general (incl. unhealthy behavior)	General health or well-being (incl. morbidity, mortality)
Cavill [72]	2020	Causal loop diagram	Literature, author expertise, and stakeholder consultations	Combination of specific SEP indicators	Physical activity or sedentary behavior	Chronic disease
Chastin [56]	2016	Conceptual framework	Literature review	Combination of specific SEP indicators	Physical activity or sedentary behavior; diet or eating behavior	General health or well-being (incl. morbidity, mortality)
Crielaard [71]	2021	Causal loop diagram	Literature, author expertise, and stakeholder consultations	SEP (in general)	None	None
De Viron [57]	2013	Conceptual model	Iterative model building process based on existing models, discussion, and re-examination by the authors	Combination of specific SEP indicators	Smoking behavior; physical activity or sedentary behavior	General health or well-being (incl. morbidity, mortality)
Dover [58]	2016	Choice set/choice transition conceptual diagram	Literature and author expertise (not explicitly stated)	Combination of specific SEP indicators	Diet or eating behavior	None
Fisher [32]	2014	Complexity framework	Literature review	Combination of specific SEP indicators	Health behavior in general (incl. unhealthy behavior)	General health or well-being (incl. morbidity, mortality); Chronic disease; Mental health outcomes
Friel [59]	2017	Causal loop diagram	Collaborative conceptual modelling	Combination of specific SEP indicators	Diet or eating behavior	None
Joffe [60]	2007	Conceptual framework	Not stated	Combination of specific SEP indicators	Hygiene practices; Diet or eating behavior	General health or well-being (incl. morbidity, mortality); Chronic disease
Majowicz [61]	2016	Conceptual systems map	Modified thematic synthesis (systematic search, inductive thematic analysis, mapping)	SEP (in general)	Diet or eating behavior	General health or well-being (incl. morbidity, mortality); Obesity
Mills [68]	2023	Causal loop diagram	Literature and stakeholder consultation	Occupation or employment	Smoking behavior	Mental health outcomes
Neff [62]	2009	Conceptual model	Literature, author expertise, and snowballing	SEP (in general)	Diet or eating behavior	General health or well-being (incl. morbidity, mortality)
Picard [63]	2011	Conceptual framework	Literature	SEP (in general)	Health behavior in general (incl. unhealthy behavior)	General health or well-being (incl. morbidity, mortality)
Rahmani [64]	2021	Conceptual framework	Literature review and expert consultation	Combination of specific SEP indicators	Diet or eating behavior; physical activity or sedentary behavior	Mental health outcomes
Reumers [69]	2022	Causal loop diagram	Literature, group model building sessions with stakeholders	Combination of specific SEP indicators	Health behavior in general (incl. unhealthy behavior); care-seeking behavior	General health or well-being (incl. morbidity, mortality); mental health outcomes
Sawyer [35]	2021	Causal loop diagram	Systematic umbrella review, expert panel, and iterative model building process	Income or economic level	Diet or eating behavior	Body mass index or body weight
Sturmberg [65]	2017	Multi-level complex adaptive systems framework	Literature and author expertise (not explicitly stated)	Combination of specific SEP indicators	None	Chronic disease
Weiler [66]	2015	Conceptual framework	Literature, intuition, and informal networking	Income or economic level	None	General health or well-being (incl. morbidity, mortality)
Wittenborn [67]	2015	Causal loop diagram	Structured umbrella review and expert consultation	Income or economic level	Sleep behavior; physical activity or sedentary behavior	General health or well-being (incl. morbidity, mortality); Chronic disease
Zukeran [70]	2017	Conceptual framework	Literature (not always explicitly stated)	Combination of specific SEP indicators	Diet or eating behavior; physical activity or sedentary behavior; smoking behavior; sleep behavior	General health or well-being (incl. morbidity, mortality); chronic disease; body mass index or body weight; mental health outcomes
*Studies containing a simulation model*						
Auchincloss [38]	2011	Agent-based model	Hypothetical relationships with some basis in theory	Income or economic level	Diet or eating behavior	None
Blok [39]	2015	Agent-based model	Literature and author expertise	Income or economic level	Diet or eating behavior	None
Blok [40]	2018	Agent-based model	Literature and author expertise	Income or economic level	Physical activity or sedentary behavior	General health or well-being (incl. morbidity, mortality)
Brittin [41]	2015	System dynamics model	Literature review	Combination of specific SEP indicators	None	Chronic disease
Broomhead [29]	2021	Agent-based model	Model structure based on literature and theory, model parametrized using empirical data	Combination of specific SEP indicators	Diet or eating behavior; hygiene practices; care-seeking behavior	General health or well-being (incl. morbidity, mortality); oral health
Chen [33]	2018	System dynamics model	Model structure basis not stated, simulation model parameterized using empirical data	Combination of specific SEP indicators	None	Body mass index or body weight
Holder [42]	1987	System dynamics model	Literature and empirical data	Income or economic level	Alcohol consumption	None
Homa [34]	2015	Agent-based model	Group model-building sessions	SEP (in general)	Health behavior in general (incl. unhealthy behavior); care-seeking behavior	General health or well-being (incl. morbidity, mortality); Chronic disease; Mental health outcomes
Li [51]	2018	Agent-based model	Model structure basis not stated, model parameterized using empirical data	Education	Diet or eating behavior	None
Lymer [43]	2012	Dynamic microsimulation	Model structure basis not stated, model parameterized using empirical data	Combination of specific SEP indicators	Physical activity or sedentary behavior; alcohol consumption; smoking behavior; care-seeking behavior	General health or well-being (incl. morbidity, mortality); Obesity
Mahamoud [44]	2013	System dynamics model	Model structure based on a participatory and iterative modelling approach, model parameterized using empirical data	Income or economic level	Health behavior in general (incl. unhealthy behavior)	General health or well-being (incl. morbidity, mortality); Chronic disease
Milstein [54]	2010	System dynamics model	Insights from previous research and stakeholder review	SEP (in general)	Health behavior in general (incl. unhealthy behavior)	General health or well-being (incl. morbidity, mortality); Chronic disease
Mooney [53]	2022	Agent-based model	Model structure basis not stated, model parameterized using empirical data	Combination of specific SEP indicators	Alcohol consumption	Chronic disease; mental health outcomes
Occhipinti [45]	2021	System dynamics model	Model structure based on iterative participatory modelling, model parameterized using empirical data	Occupation or employment	Suicidal behavior	Mental health outcomes
Orr [46]	2014	Agent-based model	Model structure basis not stated, model parameterized using literature (not explicitly stated)	Combination of specific SEP indicators	Diet or eating behavior	Body mass index or body weight; Cardiovascular disease
Orr [47]	2016	Agent-based model	Model structure basis not stated, model parameterized using literature	Combination of specific SEP indicators	Physical activity or sedentary behavior; diet or eating behavior	Body mass index or body weight
Salvo [52]	2021	Agent-based model	Model structure and parametrization based on empirical data, theory, and team expertise	Combination of specific SEP indicators	Diet or eating behavior	None
Yang [48]	2011	Agent-based model	Model structure basis not stated, model parameterized using population data	SEP (in general)	Physical activity or sedentary behavior	None
Yang [36]	2015	Agent-based model	Literature and an existing model (not explicitly stated)	Income or economic level	Physical activity or sedentary behavior	None
Yang [37]	2019	Agent-based model	Model structure adapted from existing models, model parameterized using empirical data	Income or economic level	Physical activity or sedentary behavior	General health or well-being (incl. morbidity, mortality); Mental health outcomes
Zhang [49]	2014	Agent-based model	Model structure based on theory (a multilevel theory of population health), model parameterized using literature and empirical data	Education	Diet or eating behavior	None
Zhang [50]	2018	Agent-based model	Model structure based on existing models, model parameterized using empirical data	Income or economic level	Health behavior in general (incl. unhealthy behavior)	General health or well-being (incl. morbidity, mortality)

*SEP* Socioeconomic position

A wide variety of health and health behavior outcomes were considered in the included studies, though some were more commonly modelled than others. Out of 42 total studies, 37 [29, 32, 34–40, 42–64, 67–70, 72] modelled one or more health behaviors. Diet or eating behavior (*N* = 17) [29, 35, 38, 39, 46, 47, 49, 51, 52, 56, 58–62, 64, 70] and physical activity or sedentary behavior (*N* = 12) [36, 37, 40, 43, 47, 48, 56, 57, 64, 67, 70, 72] were the most commonly modelled health behaviors. Other behaviors, including smoking (*N* = 4) [43, 57, 68, 70], alcohol consumption (*N* = 4) [42, 43, 53, 57], and sleep behavior (*N* = 2) [67, 70] were less commonly modelled. 30 studies [29, 32–35, 37, 41, 43–47, 50, 53–57, 60–70, 72] modelled one or more health outcomes, with general outcomes like health or well-being (*N* = 19) [29, 32, 34, 37, 43, 44, 50, 54–57, 60–63, 66, 67, 69, 70], mental health outcomes (including stress) (*N* = 12) [32, 34, 35, 37, 45, 53, 57, 64, 67–70], and chronic disease (*N* = 10) [34, 41, 44, 53, 54, 60, 65, 67, 70, 72] the most common. Obesity, cardiovascular disease, and oral health were present in the literature, but these were the least frequently modelled health outcomes [29, 43, 46, 61].

In addition to the categories of determinants included in the CSDH framework, individual-level determinants of behavior change (e.g., psychosocial factors, knowledge, skills, attitudes, and preferences) and the economic environment (external economic factors influencing consumers and businesses) were used to organize model elements. Overall, 41 out of 42 models (all except [33]) included at least one measure of intermediate determinants of health. These intermediate determinants included the health care system (*N* = 16) [29, 32, 34, 41, 43–45, 50, 54, 55, 57, 60, 61, 65, 68, 69], material circumstances (*N* = 37) [29, 32, 34–54, 56–62, 64–66, 68–70, 72], behaviors (*N* = 38; also includes behavior not directly related to health) [29, 32, 34–40, 42–64, 66–70, 72], individual-level determinants of behavior change (*N* = 27) [29, 34–40, 48–52, 56–61, 63, 64, 67–72], biological factors (*N* = 21) [32, 34, 37, 40, 48–50, 53, 57, 60, 61, 63–65, 67, 68, 70–72], and social cohesion (*N* = 26) [29, 32, 35–37, 40, 41, 44–49, 57, 60, 61, 63–72]. The most commonly included measures of material circumstances were related to the general physical environment (*N* = 20) [32, 35–37, 42, 48, 50, 54, 56, 58–61, 64–66, 68–70, 72], finance-related circumstances (*N* = 17) [32, 35–40, 42, 49, 51, 52, 59–61, 64, 68, 69], and the food environment (*N* = 16) [29, 32, 35, 38, 39, 42, 46, 47, 49, 51, 52, 59, 61, 62, 65, 66]. In addition to SEP, other measures of social stratification considered in the models were sex and gender (*N* = 13) [32, 37, 40, 43, 44, 48, 49, 51, 53, 57, 61, 64, 70] and ethnicity (*N* = 4) [32, 44, 52, 61]. Overall, 16 [32, 35, 42, 55–63, 66, 68, 69, 72] out of 42 models (38.1%) included at least one measure of structural determinants of health. These included governance (*N* = 2) [32, 61], macroeconomic (*N* = 5) [56, 57, 59, 61, 66], social (*N* = 2) [32, 69], and public (*N* = 6) [32, 42, 57–59, 68] policies, the economic environment (*N* = 5) [35, 59, 61, 68, 69], and culture and societal norms and values (*N* = 14) [32, 35, 42, 55, 56, 59–63, 66, 68, 69, 72].

We assessed the quality of reporting on the evidence complex systems models were based on and the extent to which key concepts of a complex systems approach were applied. About half (*N* = 23) [29, 34, 35, 37, 39–42, 44, 49, 52, 54, 56, 57, 59, 61, 63, 64, 67–69, 71, 72] of the included studies clearly described how the modelled relationships were based on literature, empirical study, or iterative model building processes. Table 1 contains descriptions of the evidence each model was based on.

**Fig. 3:**
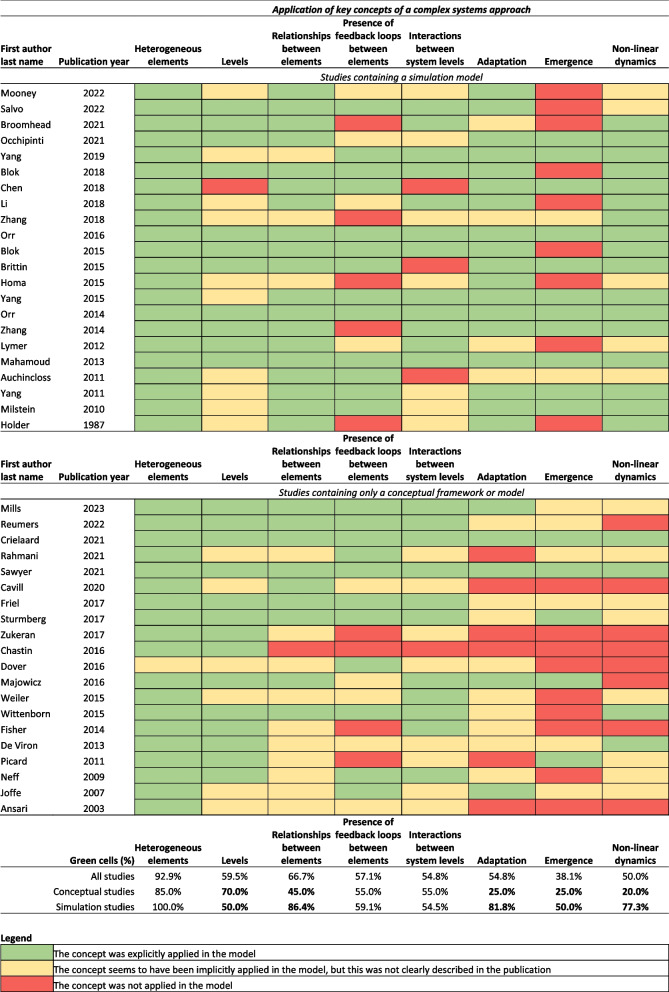
Quality assessment of the application of key concepts a systems approach

The extent to which the models in the included studies applied key concepts of a complex systems approach is shown in Fig. 3. All but one model [58] explicitly contained heterogeneous elements. Other key concepts of a complex systems approach were explicitly applied by between 38.1% (emergence) and 66.7% (relationships between elements) of studies. There were no discernable patterns in the extent to which key concepts of a complex systems approach were applied in terms of health behaviors and health outcomes included in the models or in terms of study publication dates. Studies that applied all key concepts of a complex systems approach were more likely to report polarity of the model relationships than studies that did not apply at least one key concept (80.0% vs. 53.8%).

### A visualization of the current state of research in a summary conceptual systems map

The direction of relationships was reported in 36 studies [29, 33–49, 51–55, 58–62, 64–69, 71, 72], polarity was reported in 26 studies [29, 33, 35–42, 44, 46–49, 51, 52, 54, 59, 60, 64, 67–69, 71, 72], and magnitude was reported in 10 studies [29, 33, 38–42, 44, 49, 51]. Simulation models contained more detail about the modelled relationships than the conceptual systems models, though conceptual systems models were not expected to report the magnitude of relationships (direction: 95% vs. 75%, polarity: 77% vs. 45%, magnitude: 45% vs. 0%). Relationships between model elements were extracted from most studies (*N* = 35), though relationships were too vague to extract from 7 studies containing conceptual systems models (17%) [32, 36, 56–58, 63, 70]. For example, the conceptual framework presented by Chastin et al. [56] contains a list of determinants of sedentary behavior belonging to different levels of influence, but it was not possible to extract specific relationships between these determinants or levels of influence.

**Fig. 4:**
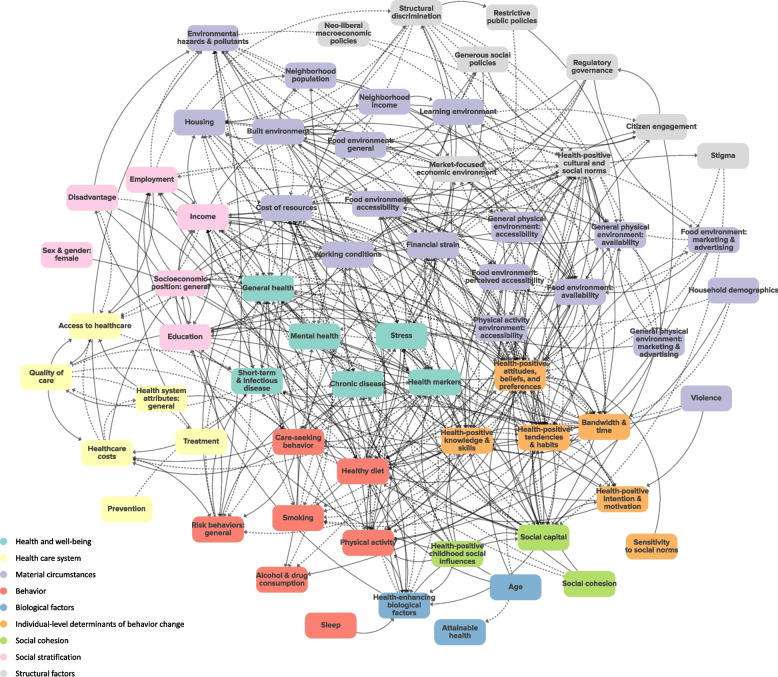
Summary conceptual systems map of complex systems research on socioeconomic inequalities in health and health behavior Solid arrows represent relationships with positive polarity, and dashed arrows represent relationships with negative polarity. We encourage readers to view an interactive version of the figure at https://kumu.io/amudd/mudd-et-al-2024-summary-conceptual-systems-map-public

The summary conceptual systems map of complex systems research on socioeconomic inequalities in health and health behavior, shown in Fig. 4, contains 66 elements and 399 relationships between these elements. The elements in the summary conceptual systems map are the element groups derived from the categorization process. The map includes relationships for which polarity was specified and consistent in the literature (positive or negative) but does not show relationships for which polarity was unspecified or inconsistent in the literature. Focusing on this subset of the relationships represented in the literature narrows the scope of the summary conceptual systems map in a way that favors studies that applied the key concepts of a complex systems approach, since the models in these studies were more likely to report relationship polarity. Figure 4 visualizes the complexity and interrelatedness of elements at different levels of influence, and the numerous displayed elements belonging to the material circumstances category illustrate the prominence of material circumstances in the literature. For more detailed insights, we encourage readers to view an interactive version of the summary conceptual systems map on the Kumu website: https://kumu.io/amudd/mudd-et-al-2024-summary-conceptual-systems-map-public [73]. An interactive version of the map that includes the additional 400 relationships for which polarity could not be deduced or was conflicting in our analysis can also be found on the Kumu website. The interactive version includes functionalities such as zooming in on the full map, zooming in on specific model elements (and their relationships with other elements), and filtering based on type of element or relationship. Supplementary File 4 lists the references for all relationships in the summary conceptual systems map and for relationships with unspecified or conflicting polarity, which are only visible in the interactive version of the summary conceptual systems map.

Social capital, income, financial strain, the built environment, and health-positive attitudes, beliefs, and preferences were direct drivers of the largest number of other elements in the summary conceptual systems map, meaning that these elements had the most outgoing arrows towards other elements. Health-positive attitudes, beliefs, and preferences, health-positive tendencies and habits, general health, and healthy diet had the most incoming arrows from other elements, meaning that they were directly driven by the largest number of other elements in the summary conceptual systems map.

**Fig. 5:**
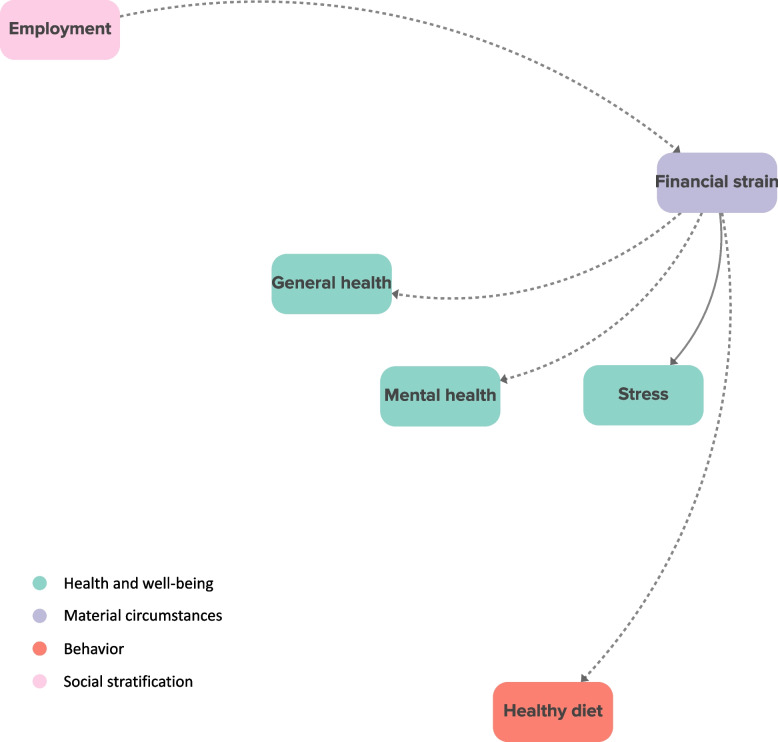
Financial strain as a shared driver of socioeconomic inequalities in multiple health and health behavior outcomes Solid arrows represent relationships with positive polarity, and dashed arrows represent relationships with negative polarity

We identified 15 direct shared drivers of socioeconomic inequalities in health and health behavior, many of which were material circumstances (*N* = 4) or individual-level determinants of behavior change (*N* = 4). General health, financial strain, the cost of resources, healthy diet, and health-positive attitudes, beliefs, and preferences were direct shared drivers of socioeconomic inequalities in the greatest number of outcomes. For example, financial strain was a direct shared driver of employment-based inequalities in general health, mental health, stress, and healthy diet, which is depicted in Fig. 5. In the literature, better employment led to less financial strain, and more financial strain led to worse general and mental health, more stress, and a less healthy diet.

## Discussion

### Summary of main findings

In this systematic scoping review, we identified 42 simulation and conceptual systems models that shed light on socioeconomic inequalities in a wide variety of health and health behavior outcomes from a complex systems perspective. CLDs and agent-based models were the most frequently employed types of conceptual and simulation models, respectively. Diet and physical activity were the most commonly modelled health behaviors, and general health outcomes (like well-being and mortality) were more commonly modelled than more specific health outcomes (such as obesity and cardiovascular disease). We used the CSDH framework to categorize all elements included in the models; nearly all models contained intermediary determinants (e.g., material circumstances, social cohesion), and less than half of the models contained structural determinants (e.g., governance, social policy). Authors in about half of the included studies clearly described the evidence (literature, empirical data, stakeholders, or a combination of these) their models were based on. Based on our summary conceptual systems map, which is a visualization of the relationships modelled in the studies included in the review, we identified 15 direct shared drivers of socioeconomic inequalities in health and health behavior outcomes. Many of these shared drivers were material circumstances or individual-level determinants of behavior change.

### Main insights from our analysis of the literature

Several shared drivers of socioeconomic inequalities in multiple health and health behavior outcomes were identified in the complex systems literature. These findings, depicted in our summary conceptual systems map, show how multiple health and health behavior outcomes occur simultaneously, share common drivers, and interact with each other to produce complex health-related outcomes. The shared drivers we identified lend support to the concept of the Global Syndemic [74], which refers to co-occurring pandemics, such as obesity, undernutrition, and climate change, that impact most people across the globe. The Global Syndemic is a global application of syndemic theory, which traditionally highlights the emergence and persistent clustering of multiple conditions in the local context [75, 76]. As the studies included in our review pertained to populations of high-income countries, we can say that our summary and analysis of the model relationships revealed common drivers of a syndemic in high-income countries.

In our analysis, direct shared drivers represent the shortest causal pathways between SEP and health and health behavior outcomes. As such, according to the literature, these direct shared drivers may be efficient mechanisms to intervene on. For example, reducing financial strain for those in unfavorable employment situations may have a direct, health-positive impact on health and health behavior. The factors that drove the largest number of other factors in the literature, on the other hand, may be the most impactful to intervene on, as they influence many factors in the system. Financial strain was both a direct shared driver of socioeconomic inequalities in health and health behavior and one of the factors that drove the largest number of other factors, so alleviating financial strain for those in lower socioeconomic circumstances may be both impactful and effective at reducing these inequalities.

Diet-related behaviors were a common focus of the models in the studies included in the review. This could be because socioeconomic inequalities in diet are difficult to tackle, especially in settings where the food environment makes unhealthy foods the more accessible option, in terms of both ease and affordability [77]. Complex systems may therefore be an especially helpful approach to understanding socioeconomic inequalities in diet, and the food environment may be considered an important driver of these inequalities by researchers. Another, non-mutually exclusive possibility, is that complex systems methods are more prevalent in the food and diet research community. The agent-based model of income inequalities in diet by Auchincloss et al. [38] was cited by many other studies included in this review, suggesting that it may have set an early example of using a complex systems approach for diet researchers.

Other health behaviors and outcomes were less well represented in the complex systems literature. Cardiovascular disease was included in one model, and obesity was included in two models. While cardiovascular disease remains the leading cause of death worldwide [78, 79], deaths due to cardiovascular disease in high income countries have decreased in the last twenty years, leading to more diversity in causes of death, including deaths from Alzheimer’s disease, cancer, and kidney disease [80]. This shift may explain why researchers modelled general health and well-being outcomes more often than specific health outcomes, as causal pathways through specific diseases may not have been considered influential in the complex system as a whole. Another possible explanation for this shift is that researchers were more focused on better understanding how determinants at other levels of influence, such as material circumstances, influenced the complex system underlying socioeconomic inequalities in health (including feedback loops between general health outcomes and fundamental underlying causes) rather than investigating specific health outcomes. Certain health behaviors, including smoking, alcohol consumption, and sleep, were also less present than expected in the reviewed literature. For example, sleep was considered in two models but was only directly related to health-enhancing biological factors in the summary conceptual systems map. Although poor sleep is associated with several chronic illnesses, socioeconomic inequalities in sleep have primarily been studied using cross-sectional cohort analysis approaches [81], so sleep seems like an area that could benefit from further research from a complex systems perspective.

Complex systems model structures are influenced by the way the model developers view the world [71], which may explain why structural determinants were considered less often than intermediate determinants like social cohesion. In the CSDH framework, the structural determinants of health are defined as key institutions and processes of the political and socioeconomic context [27]. Because the studies in this review were required to include a measure of SEP, all studies accounted for the socioeconomic context. Culture and societal norms and values were the most commonly considered structural determinants (included in a third of the models), but other determinants more explicitly related to key institutions, such as governance and social policies, were seldom present in the models. Reumers et al. acknowledged the impact of the model developers’ perspectives on the causal structure of their causal loop diagram about the health effects of social determinants of health [69]. In interviews, the policymakers and practitioners who developed the model understood the importance of the influence of structural determinants on health, but they viewed these determinants as external factors beyond their means of change. In many other studies, the position of the model developers and researchers, and its impact on the causal structure of the complex systems they depict, may not be explicitly acknowledged.

### Methodological considerations

Some of our efforts to narrow the scope of the review have implications for the interpretation of our findings. We focused on peer-reviewed literature published in scientific journals, which means that we did not include other complex systems models of socioeconomic inequalities in health (e.g., [82, 83]). Focusing on studies that self-reported applying a complex systems approach was a deliberate choice, given the aim of our review to summarize this growing body of research. As the findings from the quality assessment show, however, key concepts of a complex systems approach were not applied consistently in the studies included in the review. It is also possible that our review did not capture studies that applied key concepts of a complex systems approach without reporting doing so.

Other methodological considerations relate to our approach to synthesizing the relationships included in the 42 studies in the review. The summary conceptual systems map built for this study is a reflection of the current state of research on socioeconomic inequalities in health and health behavior mapped out from a complex systems perspective, an amalgamation of how other researchers mapped the causal structures of the complex systems they set out to model. While we can say that there is evidence that the identified influential mechanisms and shared drivers play a role in the complex system, we cannot discount the importance of determinants and relationships not (yet) depicted in the literature.

By using the existing CSDH framework and a systematic method, the summary conceptual systems map is an intuitive and insightful visualization of the current state of the literature. An important limitation of this approach, however, is that it may overgeneralize relationships between factors intended to apply to specific contexts. Understanding the context appears to be essential for altering specific systems in a meaningful way [84]. That said, by focusing on models of the adult general population in high-income countries, we hope to have captured mechanisms that are relevant to this relatively broad population, and we argue that any attempt to summarize this research would forfeit some detail.

### Implications for future research

Complex systems and systems thinking are increasingly popular topics in the public health research community. This popularity was exemplified in our review, as many studies mentioned complex systems, usually in their introduction or discussion, but did not apply the approach themselves (these studies were excluded in the full text phase of the review). There appears to be an awareness of the importance of systems thinking, but it is possible that researchers lack the knowledge or confidence required to apply a complex systems approach in their own work [11, 85]. Our review summarizes the peer-reviewed literature in which a complex systems approach was applied, which could serve as a helpful starting point for researchers seeking to understand the topic or apply a complex systems approach themselves.

We envision two main ways that our summary conceptual systems map could be useful for further research. The first is to develop a complex systems model for a specific context, such as a specific population, geographical area, or narrower scope. Model elements could be expanded on, adapted, or removed depending on expertise from stakeholders to represent the complexities of that specific context, while ensuring that model developers take elements that they may not have considered otherwise, such as structural determinants, into account. Our summary conceptual systems map could also be used as a starting point for developing a general framework for socioeconomic inequalities in health and health behavior from a complex systems perspective, an approach that has previously been applied to global cancer disparities [86]. Developing such a framework should be done in collaboration with researchers, policymakers, and members of the population with varying socioeconomic backgrounds through an iterative process such as group model building [87]. The value of a framework for understanding socioeconomic inequalities in health and health behavior from a complex systems perspective would complement existing linear frameworks and could serve as a strong underpinning for future research and policy on socioeconomic inequalities in health.

## Conclusions

In this systematic scoping review, we summarized what is currently known about socioeconomic inequalities in health and health behavior from a complex systems perspective. We visualized the content of existing models in a summary conceptual systems map, showing the interconnectedness of SEP, multiple health behaviors and health outcomes, and determinants of socioeconomic inequalities in health. Certain factors, such as financial strain, were identified as central in the summary conceptual systems map and may be especially efficient and effective policy levers to reduce socioeconomic inequalities in multiple health and health behaviors. Diet and physical activity, general health outcomes, and intermediary determinants (e.g., material circumstances) were studied relatively often from a complex systems perspective. Other mechanisms, including certain health behaviors (e.g., sleep) and structural determinants (e.g., governance), may warrant more attention. Our systematic, comprehensive synthesis of the current state of complex systems research on socioeconomic inequalities in health and health behavior may be a helpful starting point for future research on socioeconomic inequalities in health.

### Supplementary Information

**Additional file 1. **PRISMA checklist.

**Additional file 2. **Full search strategy.

**Additional file 3. **Overview of quality assessments.

**Additional file 4. **List of relationships included in the summary conceptual systems map.

## Data Availability

The full search strings are available in Supplementary File 2; key model and study characteristics are presented in Table 1; findings from the quality assessments are available in Table 1 and Fig. 3; and the data used to populate the summary conceptual systems map are available in Supplementary File 4. Data on all model elements and relationships extracted from the studies included in the review are available upon reasonable request.

## Abbreviations

CLD: Causal loop diagram
CSDH: Commission on Social Determinants of Health
OECD: Organization for Economic Co-operation and Development
SEP: Socioeconomic position
