# Supplementary material for: The current state of complex systems research on socioeconomic inequalities in health and health behavior—a systematic scoping review
Source: Int J Behav Nutr Phys Act. 2024 Feb 5;21:13. doi: 10.1186/s12966-024-01562-1 (PMC10845451; doi:10.1186/s12966-024-01562-1)
Supplement: Supplementary file 2 — Additional file 2. Full search strategy. [file 12966_2024_1562_MOESM2_ESM.docx]

# Supplementary file 2: Full search strategy

**Embase.com**

(socioeconomics/de OR 'educational status'/de OR 'economic status'/de OR 'household economic status'/de OR 'social status'/exp OR 'income group'/exp OR employment/de OR 'employment status'/exp OR income/de OR 'social environment'/de OR 'social determinants of health'/de OR (socioeconomic* OR socio-economic* OR (social NEAR/3 (status OR class OR background OR environment* OR determinant* OR context*)) OR income* OR poverty* OR wealth OR employment* OR unemployment* OR employab* OR ((occupation* OR job OR work) NEAR/3 status) OR disadvantag* OR ses OR sep):ab,ti,kw) AND (morbidity/exp OR 'chronic disease'/exp OR ('self evaluation'/de AND health/de) OR 'health status'/de OR 'health disparity'/de OR 'social determinants of health'/de OR 'health inequality'/de OR 'health inequity'/de OR 'health equity'/de OR 'cardiovascular disease'/exp OR neoplasm/exp OR 'oncological parameters'/exp OR 'diabetes mellitus'/exp OR obesity/exp OR 'lung disease'/exp OR 'health behavior'/de OR 'tobacco use'/exp OR diet/de OR 'dietary intake'/exp OR 'physical activity'/exp OR exercise/de OR sport/de OR 'alcohol abuse'/exp OR alcoholism/de OR 'drinking behavior'/de OR 'food intake'/de OR 'vegetable consumption'/de OR 'fruit consumption'/de OR (morbidit* OR (health NEAR/3 (outcome* OR disparit* OR inequalit* OR inequit* OR equalit* OR equit* OR determinant*)) OR ((chronic OR heart OR cardiac) NEAR/3 (disease* OR condition*)) OR (self NEXT/1 (assess* OR perceiv* OR report*) NEAR/3 (health*)) OR cardiovascul* OR cardio-vascul* OR ((myocardial OR heart) NEAR/3 (infarct*)) OR neoplasm* OR cancer* OR diabet* OR obes* OR ((lung OR pulmonar*) NEAR/3 disease*) OR copd OR ((health* OR unhealth*) NEAR/3 (behav* OR status)) OR (tobacco NEAR/3 use) OR smoking OR diet* OR ((fruit* OR vegetable* OR food OR alcohol OR beverage*) NEAR/3 (consumption* OR intake*)) OR ((physical OR behav*) NEAR/3 (activ* OR inactiv*)) OR sedentar* OR exercis* OR exerciz* OR (sport* NEAR/3 (participat*)) OR (alcohol NEAR/3 (abuse OR use)) OR (drinking NEAR/3 (heavy OR excessive OR behav*)) OR stroke* OR cva OR ((cerebrovasc* OR cerebro-vasc*) NEAR/3 accident*) OR alcoholism ):Ab,ti,kw) AND (microsimulation/exp OR 'agent based model'/de OR 'system dynamics model'/de OR 'differential equation'/de OR 'differential equation model'/de OR 'systems theory'/de OR 'system dynamics'/de OR (('conceptual model'/de OR 'conceptual framework'/de OR 'theoretical model'/de) AND (system:ab,ti,kw)) OR 'agent based modeling'/de OR 'micro simulation model'/de OR 'system dynamics modeling'/de OR (microsimulat* OR micro-simulat* OR ((agent-based OR system*-dynamic* OR complex OR system OR systems) NEAR/3 (model* OR framework* OR frame-work*)) OR ((conceptual* OR theoretic*) NEAR/3 (model* OR framework* OR frame-work*) AND (system OR systems*)) OR (( systems OR system) NEAR/3 (theory OR dynamic*)) OR differen*-equation*):ab,ti,kw) NOT ([animals]/lim NOT [humans]/lim) NOT ([conference abstract]/lim AND [2000-2018]/py) AND [english]/lim

**Medline ALL Ovid**

(Socioeconomic Factors/ OR Educational Status/ OR Economic Status/ OR Employment/ OR Income/ OR Poverty/ OR Social Environment/ OR Social Determinants of Health/ OR (socioeconomic* OR socio-economic* OR (social ADJ3 (status OR class OR background OR environment* OR determinant* OR context*)) OR income* OR poverty* OR wealth OR employment* OR unemployment* OR employab* OR ((occupation* OR job OR work) ADJ3 status) OR disadvantag* OR ses OR sep).ab,ti,kf.) AND (Morbidity / OR Chronic Disease / OR (Self-Assessment / AND Health /) OR Diagnostic Self Evaluation / OR Health Status / OR Health Status Indicators / OR Health Status Disparities / OR Social Determinants of Health / OR Health Equity / OR Cardiovascular Diseases / OR exp Neoplasms/ OR Diabetes Mellitus / OR Obesity / OR exp Lung Diseases / OR Health Behavior / OR exp "Tobacco Use"/ OR exp Diet / OR Exercise / OR Sports/ OR Alcoholism / OR exp Drinking Behavior / OR Eating / OR (morbidit* OR (health ADJ3 (outcome* OR disparit* OR inequalit* OR inequit* OR equalit* OR equit* OR determinant*)) OR ((chronic OR heart OR cardiac) ADJ3 (disease* OR condition*)) OR (self ADJ (assess* OR perceiv* OR report*) ADJ3 (health*)) OR cardiovascul* OR cardio-vascul* OR ((myocardial OR heart) ADJ3 (infarct*)) OR neoplasm* OR cancer* OR diabet* OR obes* OR ((lung OR pulmonar*) ADJ3 disease*) OR copd OR ((health* OR unhealth*) ADJ3 (behav* OR status)) OR (tobacco ADJ3 "use") OR smoking OR diet* OR ((fruit* OR vegetable* OR food OR alcohol OR beverage*) ADJ3 (consumption* OR intake*)) OR ((physical OR behav*) ADJ3 (activ* OR inactiv*)) OR sedentar* OR exercis* OR exerciz* OR (sport* ADJ3 (participat*)) OR (alcohol ADJ3 (abuse OR "use")) OR (drinking ADJ3 (heavy OR excessive OR behav*)) OR stroke* OR cva OR ((cerebrovasc* OR cerebro-vasc*) ADJ3 accident*) OR alcoholism ).ab,ti,kf.) AND (Systems Theory/ OR Systems Analysis/ OR (microsimulat* OR micro-simulat* OR ((agent-based OR system*-dynamic* OR complex OR system OR systems) ADJ3 (model* OR framework* OR frame-work*)) OR ((conceptual* OR theoretic*) ADJ3 (model* OR framework* OR frame-work*) AND (system OR systems*)) OR (( systems OR system) ADJ3 (theory OR dynamic*)) OR differen*-equation*).ab,ti,kf.) NOT (exp animals/ NOT humans/) NOT ((news OR congres* OR abstract* OR book* OR chapter* OR dissertation abstract*).pt. AND 1800:2018.(sa_year).) AND english.la.

**Web of Science Core Collection**

TS=(((socioeconomic* OR socio-economic* OR (social NEAR/2 (status OR class OR background OR environment* OR determinant* OR context*)) OR income* OR poverty* OR wealth OR employment* OR unemployment* OR employab* OR ((occupation* OR job OR work) NEAR/2 status) OR disadvantag* OR ses OR sep)) AND ((morbidit* OR (health NEAR/2 (outcome* OR disparit* OR inequalit* OR inequit* OR equalit* OR equit* OR determinant*)) OR ((chronic OR heart OR cardiac) NEAR/2 (disease* OR condition*)) OR (self NEAR/1 (assess* OR perceiv* OR report*) NEAR/2 (health*)) OR cardiovascul* OR cardio-vascul* OR ((myocardial OR heart) NEAR/2 (infarct*)) OR neoplasm* OR cancer* OR diabet* OR obes* OR ((lung OR pulmonar*) NEAR/2 disease*) OR copd OR ((health* OR unhealth*) NEAR/2 (behav* OR status)) OR (tobacco NEAR/2 use) OR smoking OR diet* OR ((fruit* OR vegetable* OR food OR alcohol OR beverage*) NEAR/2 (consumption* OR intake*)) OR ((physical OR behav*) NEAR/2 (activ* OR inactiv*)) OR sedentar* OR exercis* OR exerciz* OR (sport* NEAR/2 (participat*)) OR (alcohol NEAR/2 (abuse OR use)) OR (drinking NEAR/2 (heavy OR excessive OR behav*)) OR stroke* OR cva OR ((cerebrovasc* OR cerebro-vasc*) NEAR/2 accident*) OR alcoholism )) AND ((microsimulat* OR micro-simulat* OR ((agent-based OR system*-dynamic* OR complex OR system OR systems) NEAR/2 (model* OR framework* OR frame-work*)) OR ((conceptual* OR theoretic*) NEAR/2 (model* OR framework* OR frame-work*) AND (system OR systems*)) OR (( systems OR system) NEAR/2 (theory OR dynamic*)) OR differen*-equation*))) NOT (DT=(Meeting Abstract OR Meeting Summary) AND py=(1800-2018)) AND LA=(english)

**CINAHL EBSCOhost**

(MH Socioeconomic Factors OR MH Educational Status OR MH Economic Status OR MH Employment OR MH Income OR MH Poverty OR MH Social Environment OR MH Social Determinants of Health OR TI(socioeconomic* OR socio-economic* OR (social N2 (status OR class OR background OR environment* OR determinant* OR context*)) OR income* OR poverty* OR wealth OR employment* OR unemployment* OR employab* OR ((occupation* OR job OR work) N2 status) OR disadvantag* OR ses OR sep) OR AB(socioeconomic* OR socio-economic* OR (social N2 (status OR class OR background OR environment* OR determinant* OR context*)) OR income* OR poverty* OR wealth OR employment* OR unemployment* OR employab* OR ((occupation* OR job OR work) N2 status) OR disadvantag* OR ses OR sep)) AND (MH Morbidity OR MH Chronic Disease OR (MH Self Assessment AND MH Health) OR MH Health Status OR MH Health Status Indicators OR MH Health Status Disparities OR MH Social Determinants of Health OR MH Cardiovascular Diseases OR MH Neoplasms+ OR MH Diabetes Mellitus + OR MH Obesity + OR MH Lung Diseases + OR MH Health Behavior OR MH Smoking+ OR MH Diet + OR MH Exercise OR MH Sports OR MH Alcoholism OR MH Drinking Behavior + OR MH Eating OR MH Eating Behavior OR TI(morbidit* OR (health N2 (outcome* OR disparit* OR inequalit* OR inequit* OR equalit* OR equit* OR determinant*)) OR ((chronic OR heart OR cardiac) N2 (disease* OR condition*)) OR (self N1 (assess* OR perceiv* OR report*) N2 (health*)) OR cardiovascul* OR cardio-vascul* OR ((myocardial OR heart) N2 (infarct*)) OR neoplasm* OR cancer* OR diabet* OR obes* OR ((lung OR pulmonar*) N2 disease*) OR copd OR ((health* OR unhealth*) N2 (behav* OR status)) OR (tobacco N2 "use") OR smoking OR diet* OR ((fruit* OR vegetable* OR food OR alcohol OR beverage*) N2 (consumption* OR intake*)) OR ((physical OR behav*) N2 (activ* OR inactiv*)) OR sedentar* OR exercis* OR exerciz* OR (sport* N2 (participat*)) OR (alcohol N2 (abuse OR "use")) OR (drinking N2 (heavy OR excessive OR behav*)) OR stroke* OR cva OR ((cerebrovasc* OR cerebro-vasc*) N2 accident*) OR alcoholism ) OR AB(morbidit* OR (health N2 (outcome* OR disparit* OR inequalit* OR inequit* OR equalit* OR equit* OR determinant*)) OR ((chronic OR heart OR cardiac) N2 (disease* OR condition*)) OR (self N1 (assess* OR perceiv* OR report*) N2 (health*)) OR cardiovascul* OR cardio-vascul* OR ((myocardial OR heart) N2 (infarct*)) OR neoplasm* OR cancer* OR diabet* OR obes* OR ((lung OR pulmonar*) N2 disease*) OR copd OR ((health* OR unhealth*) N2 (behav* OR status)) OR (tobacco N2 "use") OR smoking OR diet* OR ((fruit* OR vegetable* OR food OR alcohol OR beverage*) N2 (consumption* OR intake*)) OR ((physical OR behav*) N2 (activ* OR inactiv*)) OR sedentar* OR exercis* OR exerciz* OR (sport* N2 (participat*)) OR (alcohol N2 (abuse OR "use")) OR (drinking N2 (heavy OR excessive OR behav*)) OR stroke* OR cva OR ((cerebrovasc* OR cerebro-vasc*) N2 accident*) OR alcoholism )) AND (MH Systems Theory+ OR MH Systems Analysis+ OR TI(microsimulat* OR micro-simulat* OR ((agent-based OR system*-dynamic* OR complex OR system OR systems) N2 (model* OR framework* OR frame-work*)) OR ((conceptual* OR theoretic*) N2 (model* OR framework* OR frame-work*) AND (system OR systems*)) OR (( systems OR system) N2 (theory OR dynamic*)) OR differen*-equation*) OR AB(microsimulat* OR micro-simulat* OR ((agent-based OR system*-dynamic* OR complex OR system OR systems) N2 (model* OR framework* OR frame-work*)) OR ((conceptual* OR theoretic*) N2 (model* OR framework* OR frame-work*) AND (system OR systems*)) OR (( systems OR system) N2 (theory OR dynamic*)) OR differen*-equation*)) NOT (MH animals+ NOT MH humans+) NOT (MH News OR MH Abstracts OR MH Books+) AND LA english
