# Supplementary material for: The current state of complex systems research on socioeconomic inequalities in health and health behavior—a systematic scoping review
Source: Int J Behav Nutr Phys Act. 2024 Feb 5;21:13. doi: 10.1186/s12966-024-01562-1 (PMC10845451; doi:10.1186/s12966-024-01562-1)
Supplement: Supplementary file 3 — Additional file 3. Overview of quality assessments. [file 12966_2024_1562_MOESM3_ESM.docx]

# Supplementary file 3: Overview of quality assessments

Table 1: Overview of quality assessments for evidence models were based on and the application of a complex systems approach

| Criterium | Green light | Yellow light | Red light |
| --- | --- | --- | --- |
| Evidence base for model: | The study based the modelled relationships on literature, empirical study, or iterative model building processes that are clearly described in the publication. | The study seems to have based the modelled relationships on literature, empirical study, or iterative model building processes, but these are not clearly described in the publication. | The study did not base the modelled relationships on literature, empirical study, or iterative model building processes |
| Application of complex systems approach (each concept listed below will be assessed separately with the traffic light system): | The concept was explicitly applied in the model. | The concept seems to have been implicitly applied in the model, but this was not clearly described in the publication. | The concept was not applied in the model. |
| **Heterogeneous elements**: Distinct system elements that characterize the agents in the system | | | |
| **Levels**: A description of the system structure and the level(s) within that structure | | | |
| **Relationships between elements**: Connections or interactions between system elements | | | |
| **Presence of feedback loops between elements**: Responses between elements that may alter the intervention and its impacts. Can be reinforcing, positive, negative, or balancing | | | |
| **Interactions between system levels**: Elements at one system level influence elements at other system levels | | | |
| **Adaptation**: Adjustments in system behavior in response to internal and external change | | | |
| **Emergence**: Patterns that emerge from the interplay between factors- system-level behavior cannot be attributed to its individual parts | | | |
| **Non-linear dynamics**: Inputs into the system do not necessarily result in correspondingly sized effects in the system, and the state of the system changes over time | | | |

*Key concepts of a complex systems approach are based on: Diez Roux* (1)*, Rutter et al.* (2)*,* and the traffic light system from *McGill et al.* (3)*.*

## References

1. Diez Roux AV. Complex Systems Thinking and Current Impasses in Health Disparities Research. American Journal of Public Health. 2011;101:1627–34.

2. Rutter H, Cavill N, Bauman A, Bull F. Systems approaches to global and national physical activity plans. Bull World Health Organ. 2019 Feb 1;97(2):162–5.

3. McGill E, Marks D, Er V, Penney T, Petticrew M, Egan M. Qualitative process evaluation from a complex systems perspective: A systematic review and framework for public health evaluators. Kruk ME, editor. PLoS Med. 2020 Nov 2;17(11):e1003368.
