# Supplementary material for: The current state of complex systems research on socioeconomic inequalities in health and health behavior—a systematic scoping review
Source: Int J Behav Nutr Phys Act. 2024 Feb 5;21:13. doi: 10.1186/s12966-024-01562-1 (PMC10845451; doi:10.1186/s12966-024-01562-1)
Supplement: Supplementary file 4 — Additional file 4. List of relationships included in the summary conceptual systems map. [file 12966_2024_1562_MOESM4_ESM.docx]

# Supplementary file 4: List of relationships included in the summary conceptual systems map

Table 1: Rules for determining relationship polarity in the summary conceptual systems map

| **Polarities of relationships from specific studies** | **Polarity in summary conceptual systems map** | ***Example*** |
| --- | --- | --- |
| Specified and matching | Positive or negative | *Relationship 1 polarity: Positive*  *Relationship 2 polarity: Positive*  *Polarity in summary conceptual systems map: Positive* |
| Specified and conflicting | Conflicting | *Relationship 1 polarity: Positive*  *Relationship 2 polarity: Negative*  *Polarity in summary conceptual systems map: Conflicting* |
| Some specified and matching, some unspecified | Positive or negative | *Relationship 1 polarity: Positive*  *Relationship 2 polarity: Positive*  *Relationship 3 polarity: Unspecified*  *Polarity in summary conceptual systems map: Positive* |
| Some specified and conflicting, some unspecified | Unspecified | *Relationship 1 polarity: Positive*  *Relationship 2 polarity: Negative*  *Relationship 3 polarity: Unspecified*  *Polarity in summary conceptual systems map: Unspecified* |
| All unspecified | Unspecified | *Relationship 1 polarity: Unspecified*  *Relationship 2 polarity: Unspecified*  *Polarity in summary conceptual systems map: Unspecified* |

Table 2: List of relationships included in the summary conceptual systems map

| **From** | **From** | **To** | **Reference(s)** | **Polarity in summary conceptual systems map** |
| --- | --- | --- | --- | --- |
| *CSDH-based category* | *Element group* | *Element group* |  | *Positive, negative, conflicting, or unspecified* |
| Health and well-being | General health | General health | Joffe, 2007 (1);Lymer and Brown, 2012 (2) | Positive |
|  | General health | Chronic disease | Wittenborn et al., 2015 (3) | Negative |
|  | General health | Obesity | Majowicz et al., 2016 (4) | Unspecified |
|  | General health | Health markers | Reumers et al., 2022 (5) | Positive |
|  | General health | Health system attributes: general | Ansari et al., 2003 (6) | Unspecified |
|  | General health | Healthcare costs | Joffe, 2007 (1) | Unspecified |
|  | General health | Food environment: availability | Majowicz et al., 2016 (4) | Unspecified |
|  | General health | Financial strain | Majowicz et al., 2016 (4) | Unspecified |
|  | General health | Working conditions | Joffe, 2007 (1) | Positive |
|  | General health | Household demographics | Majowicz et al., 2016 (4) | Unspecified |
|  | General health | Healthy diet | Majowicz et al., 2016 (4) | Unspecified |
|  | General health | Physical activity | Lymer and Brown, 2012 (2); Wittenborn et al., 2015 (3); Cavill et al., 2020 (7) | Positive |
|  | General health | Alcohol & drug consumption | Lymer and Brown, 2012 (2) | Unspecified |
|  | General health | Smoking | Lymer and Brown, 2012 (2) | Unspecified |
|  | General health | Care-seeking behavior | Lymer and Brown, 2012 (2); Reumers et al., 2022 (5) | Negative |
|  | General health | Health-enhancing biological factors | Wittenborn et al., 2015 (3) | Positive |
|  | General health | Health-positive attitudes, beliefs, and preferences | Reumers et al., 2022 (5); Cavill et al., 2020 (7) | Positive |
|  | General health | Health-positive knowledge & skills | Reumers et al., 2022 (5) | Positive |
|  | General health | Social capital | Reumers et al., 2022 (5) | Positive |
|  | General health | Income | Mahamoud et al., 2013 (8) | Negative |
|  | General health | Education | Joffe, 2007 (1) | Positive |
|  | General health | Health-positive cultural and social norms | Weiler et al., 2015 (9) | Unspecified |
|  | Mental health | Mental health | Mooney et al., 2022 (10) | Unspecified |
|  | Mental health | Stress | Mooney et al., 2022 (10) | Unspecified |
|  | Mental health | Violence | Mooney et al., 2022 (10) | Unspecified |
|  | Mental health | Household demographics | Mooney et al., 2022 (10) | Unspecified |
|  | Mental health | Alcohol & drug consumption | Occhipinti et al., 2021 (11); Mooney et al., 2022 (10) | Unspecified |
|  | Mental health | Care-seeking behavior | Occhipinti et al., 2021 (11) | Unspecified |
|  | Mental health | Suicidal behavior | Occhipinti et al., 2021 (11) | Unspecified |
|  | Mental health | Social capital | Broomhead et al., 2021 (12) | Positive |
|  | Mental health | Health-positive childhood social influences | Occhipinti et al., 2021 (11) | Unspecified |
|  | Mental health | Income | Mooney et al., 2022 (10) | Unspecified |
|  | Mental health | Employment | Occhipinti et al., 2021 (11) | Unspecified |
|  | Stress | General health | Reumers et al., 2022 (5) | Negative |
|  | Stress | Mental health | Mooney et al., 2022 (10) | Unspecified |
|  | Stress | Cost of resources | Reumers et al., 2022 (5) | Positive |
|  | Stress | Violence | Mooney et al., 2022 (10) | Unspecified |
|  | Stress | Household demographics | Mooney et al., 2022 (10) | Unspecified |
|  | Stress | Alcohol & drug consumption | Mooney et al., 2022 (10) | Unspecified |
|  | Stress | Smoking | Mills et al., 2023 (35) | Positive |
|  | Stress | Health-enhancing biological factors | Wittenborn et al., 2015 (3); Crielaard et al., 2021 (14) | Negative |
|  | Stress | Bandwidth & time | Crielaard et al., 2021 (14) | Negative |
|  | Stress | Health-positive attitudes, beliefs, and preferences | Friel et al., 2017 (15) | Negative |
|  | Stress | Health-positive knowledge & skills | Crielaard et al., 2021 (14) | Negative |
|  | Stress | Income | Mooney et al., 2022 (10) | Unspecified |
|  | Stress | Health-positive cultural and social norms | Sawyer et al., 2021 (16) | Negative |
|  | Chronic disease | General health | Homa et al., 2015 (17); Mahamoud et al., 2013 (8); Cavill et al., 2020 (7) | Conflicting |
|  | Chronic disease | Mental health | Mooney et al., 2022 (10) | Unspecified |
|  | Chronic disease | Stress | Mooney et al., 2022 (10) | Unspecified |
|  | Chronic disease | Chronic disease | Sturmberg et al., 2017 (18) | Unspecified |
|  | Chronic disease | Short-term & infectious disease | Milstein et al., 2010 (19) | Positive |
|  | Chronic disease | Healthcare costs | Milstein et al., 2010 (19) | Positive |
|  | Chronic disease | Built environment | Sturmberg et al., 2017 (18) | Unspecified |
|  | Chronic disease | Violence | Mooney et al., 2022 (10) | Unspecified |
|  | Chronic disease | Neighborhood population | Mahamoud et al., 2013 (8) | Unspecified |
|  | Chronic disease | Household demographics | Mooney et al., 2022 (10) | Unspecified |
|  | Chronic disease | Housing | Sturmberg et al., 2017 (18) | Unspecified |
|  | Chronic disease | Healthy diet | Sturmberg et al., 2017 (18) | Unspecified |
|  | Chronic disease | Health-enhancing biological factors | Sturmberg et al., 2017 (18); Wittenborn et al., 2015 (3) | Negative |
|  | Chronic disease | Social capital | Sturmberg et al., 2017 (18) | Unspecified |
|  | Chronic disease | Employment | Sturmberg et al., 2017 (18) | Unspecified |
|  | Chronic disease | Education | Sturmberg et al., 2017 (18) | Unspecified |
|  | Obesity | General health | Lymer and Brown, 2012 (2) | Unspecified |
|  | Obesity | Obesity | Lymer and Brown, 2012 (2) | Unspecified |
|  | Short-term & infectious disease | General health | Joffe, 2007 (1) | Negative |
|  | Short-term & infectious disease | Obesity | Majowicz et al., 2016 (4) | Unspecified |
|  | Short-term & infectious disease | Health markers | Milstein et al., 2010 (19) | Positive |
|  | Short-term & infectious disease | Quality of care | Milstein et al., 2010 (19) | Negative |
|  | Short-term & infectious disease | Healthcare costs | Milstein et al., 2010 (19) | Positive |
|  | Short-term & infectious disease | Health-enhancing biological factors | Majowicz et al., 2016 (4) | Unspecified |
|  | Short-term & infectious disease | Disadvantage | Milstein et al., 2010 (19) | Positive |
|  | Health markers | General health | Majowicz et al., 2016 (4) | Unspecified |
|  | Health markers | Chronic disease | Broomhead et al., 2021 (12) | Positive |
|  | Health markers | Short-term & infectious disease | Joffe, 2007 (1); Majowicz et al., 2016 (4) | Positive |
|  | Health markers | Health markers | Chen et al., 2018 (20) | Positive |
|  | Health markers | Healthcare costs | Milstein et al., 2010 (19) | Negative |
|  | Health markers | Food environment: availability | Majowicz et al., 2016 (4) | Unspecified |
|  | Health markers | Financial strain | Majowicz et al., 2016 (4) | Unspecified |
|  | Health markers | Environmental hazards & pollutants | Majowicz et al., 2016 (4) | Unspecified |
|  | Health markers | Healthy diet | Broomhead et al., 2021 (12) | Positive |
|  | Health markers | Care-seeking behavior | Broomhead et al., 2021 (12) | Positive |
|  | Health markers | Other behaviors | Broomhead et al., 2021 (12) | Unspecified |
|  | Health markers | Health-enhancing biological factors | Majowicz et al., 2016 (4) | Unspecified |
|  | Health markers | Health-positive intention & motivation | Broomhead et al., 2021 (12) | Negative |
|  | Health markers | Socioeconomic position: general | Majowicz et al., 2016 (4) | Unspecified |
|  | Health markers | Employment | Chen et al., 2018 (20) | Positive |
| Health care system | Health system attributes: general | General health | Ansari et al., 2003 (6);Joffe, 2007 (1) | Unspecified |
|  | Health system attributes: general | Health system attributes: general | Milstein et al., 2010 (19) | Conflicting |
|  | Health system attributes: general | Access to healthcare | Milstein et al., 2010 (19) | Positive |
|  | Health system attributes: general | Quality of care | Milstein et al., 2010 (19) | Positive |
|  | Health system attributes: general | Healthcare costs | Milstein et al., 2010 (19) | Conflicting |
|  | Health system attributes: general | Neighborhood population | Mahamoud et al., 2013 (8) | Unspecified |
|  | Health system attributes: general | Healthy diet | Majowicz et al., 2016 (4) | Unspecified |
|  | Health system attributes: general | Care-seeking behavior | Lymer and Brown, 2012 (2); Milstein et al., 2010 (19) | Positive |
|  | Health system attributes: general | Health-positive psychosocial factors: general | Ansari et al., 2003 (6) | Unspecified |
|  | Health system attributes: general | Socioeconomic position: general | Ansari et al., 2003 (6) | Unspecified |
|  | Health system attributes: general | Health-positive cultural and social norms | Ansari et al., 2003 (6) | Unspecified |
|  | Access to healthcare | General health | Mahamoud et al., 2013 (8); Milstein et al., 2010 (19); Reumers et al., 2022 (5) | Positive |
|  | Access to healthcare | Obesity | Majowicz et al., 2016 (4) | Unspecified |
|  | Access to healthcare | Short-term & infectious disease | Majowicz et al., 2016 (4) | Unspecified |
|  | Access to healthcare | Access to healthcare | Mills et al., 2023 (35) | Positive |
|  | Access to healthcare | Quality of care | Milstein et al., 2010 (19) | Positive |
|  | Access to healthcare | Natural environment | Majowicz et al., 2016 (4) | Unspecified |
|  | Access to healthcare | Financial strain | Majowicz et al., 2016 (4) | Unspecified |
|  | Access to healthcare | Environmental hazards & pollutants | Majowicz et al., 2016 (4) | Unspecified |
|  | Access to healthcare | Household demographics | Majowicz et al., 2016 (4) | Unspecified |
|  | Access to healthcare | Smoking | Mills et al., 2023 (35) | Negative |
|  | Treatment | Health-enhancing biological factors | Sturmberg et al., 2017 (18) | Unspecified |
|  | Treatment | Employment | Brittin et al., 2015 (21) | Positive |
|  | Prevention | Chronic disease | Brittin et al., 2015 (21) | Negative |
|  | Quality of care | Short-term & infectious disease | Milstein et al., 2010 (19) | Negative |
|  | Quality of care | Health markers | Milstein et al., 2010 (19) | Positive |
|  | Quality of care | Access to healthcare | Milstein et al., 2010 (19) | Positive |
|  | Quality of care | Treatment | Homa et al., 2015 (17) | Negative |
|  | Quality of care | Quality of care | Homa et al., 2015 (17); Milstein et al., 2010 (19) | Positive |
|  | Quality of care | Healthcare costs | Milstein et al., 2010 (19) | Positive |
|  | Quality of care | Care-seeking behavior | Milstein et al., 2010 (19) | Negative |
|  | Healthcare costs | Health system attributes: general | Milstein et al., 2010 (19) | Negative |
|  | Healthcare costs | Access to healthcare | Milstein et al., 2010 (19) | Negative |
|  | Healthcare costs | Cost of resources | Joffe, 2007 (1); Reumers et al., 2022 (5) | Positive |
|  | Healthcare costs | Environmental hazards & pollutants | Milstein et al., 2010 (19) | Positive |
|  | Healthcare costs | Risk behaviors: general | Milstein et al., 2010 (19) | Positive |
| Material circumstances | Food environment: general | Obesity | Majowicz et al., 2016 (4) | Unspecified |
|  | Food environment: general | Food environment: general | Neff et al., 2009 (22) | Unspecified |
|  | Food environment: general | Food environment: availability | Friel et al., 2017 (15) | Positive |
|  | Food environment: general | Financial strain | Majowicz et al., 2016 (4) | Unspecified |
|  | Food environment: general | Healthy diet | Majowicz et al., 2016 (4); Neff et al., 2009 (22) | Unspecified |
|  | Food environment: general | Health-enhancing biological factors | Majowicz et al., 2016 (4) | Unspecified |
|  | Food environment: general | Health-positive knowledge & skills | Majowicz et al., 2016 (4) | Unspecified |
|  | Food environment: general | Social cohesion | Majowicz et al., 2016 (4) | Unspecified |
|  | Food environment: general | Social capital | Neff et al., 2009 (22) | Unspecified |
|  | Food environment: general | Health-positive childhood social influences | Majowicz et al., 2016 (4) | Unspecified |
|  | Food environment: general | Market-focused economic environment | Friel et al., 2017 (15) | Positive |
|  | Food environment: general | Health-positive cultural and social norms | Majowicz et al., 2016 (4) | Unspecified |
|  | Food environment: availability | Obesity | Majowicz et al., 2016 (4) | Unspecified |
|  | Food environment: availability | Short-term & infectious disease | Majowicz et al., 2016 (4) | Unspecified |
|  | Food environment: availability | Access to healthcare | Majowicz et al., 2016 (4) | Unspecified |
|  | Food environment: availability | Food environment: general | Majowicz et al., 2016 (4) | Unspecified |
|  | Food environment: availability | Food environment: availability | Auchincloss et al., 2011 (23); Blok et al., 2015 (24); Friel et al., 2017 (15); Majowicz et al., 2016 (4); Sawyer et al., 2021 (16) | Positive |
|  | Food environment: availability | Food environment: accessibility | Sawyer et al., 2021 (16) | Positive |
|  | Food environment: availability | Food environment: perceived accessibility | Sawyer et al., 2021 (16) | Positive |
|  | Food environment: availability | Food environment: marketing & advertising | Majowicz et al., 2016 (4) | Unspecified |
|  | Food environment: availability | General physical environment: marketing & advertising | Holder and Blose, 1987 (25) | Unspecified |
|  | Food environment: availability | Financial strain | Majowicz et al., 2016 (4); Sawyer et al., 2021 (16) | Negative |
|  | Food environment: availability | Cost of resources | Friel et al., 2017 (15); Sawyer et al., 2021 (16) | Conflicting |
|  | Food environment: availability | Environmental hazards & pollutants | Majowicz et al., 2016 (4) | Unspecified |
|  | Food environment: availability | Healthy diet | Friel et al., 2017 (15); Majowicz et al., 2016 (4); Orr et al., 2014 (26); Orr et al., 2016 (27); Broomhead et al., 2021 (12); Salvo et al., 2022 (28) | Positive |
|  | Food environment: availability | Health-positive tendencies & habits | Friel et al., 2017 (15); Salvo et al., 2022 (28) | Positive |
|  | Food environment: availability | Health-positive attitudes, beliefs, and preferences | Friel et al., 2017 (15); Sawyer et al., 2021 (16); Salvo et al., 2022 (28) | Positive |
|  | Food environment: availability | Health-positive knowledge & skills | Majowicz et al., 2016 (4) | Unspecified |
|  | Food environment: availability | Socioeconomic position: general | Majowicz et al., 2016 (4) | Unspecified |
|  | Food environment: availability | Income | Holder and Blose, 1987 (25) | Unspecified |
|  | Food environment: availability | Restrictive public policies | Holder and Blose, 1987 (25) | Unspecified |
|  | Food environment: availability | Market-focused economic environment | Sawyer et al., 2021 (16) | Negative |
|  | Food environment: availability | Health-positive cultural and social norms | Majowicz et al., 2016 (4) | Unspecified |
|  | Food environment: accessibility | General health | Weiler et al., 2015 (9) | Unspecified |
|  | Food environment: accessibility | Food environment: availability | Friel et al., 2017 (15) | Positive |
|  | Food environment: accessibility | Food environment: accessibility | Friel et al., 2017 (15) | Positive |
|  | Food environment: accessibility | Food environment: perceived accessibility | Friel et al., 2017 (15); Li et al., 2018 (13) | Positive |
|  | Food environment: accessibility | General physical environment: accessibility | Sawyer et al., 2021 (16) | Positive |
|  | Food environment: accessibility | Healthy diet | Auchincloss et al., 2011 (23); Blok et al., 2015 (24); Friel et al., 2017 (15); Zhang et al., 2014 (29) | Positive |
|  | Food environment: accessibility | Bandwidth & time | Friel et al., 2017 (15) | Positive |
|  | Food environment: accessibility | Health-positive attitudes, beliefs, and preferences | Sawyer et al., 2021 (16) | Positive |
|  | Food environment: accessibility | Health-positive knowledge & skills | Friel et al., 2017 (15) | Positive |
|  | Food environment: accessibility | Social capital | Friel et al., 2017 (15) | Positive |
|  | Food environment: accessibility | Regulatory governance | Weiler et al., 2015 (9) | Unspecified |
|  | Food environment: perceived accessibility | Healthy diet | Li et al., 2018 (13) | Positive |
|  | Food environment: perceived accessibility | Health-positive attitudes, beliefs, and preferences | Sawyer et al., 2021 (16) | Positive |
|  | Food environment: marketing & advertising | Obesity | Majowicz et al., 2016 (4) | Unspecified |
|  | Food environment: marketing & advertising | Food environment: general | Majowicz et al., 2016 (4) | Unspecified |
|  | Food environment: marketing & advertising | Food environment: availability | Friel et al., 2017 (15); Majowicz et al., 2016 (4) | Positive |
|  | Food environment: marketing & advertising | Food environment: perceived accessibility | Sawyer et al., 2021 (16) | Negative |
|  | Food environment: marketing & advertising | Food environment: marketing & advertising | Friel et al., 2017 (15); Sawyer et al., 2021 (16) | Positive |
|  | Food environment: marketing & advertising | Financial strain | Majowicz et al., 2016 (4) | Unspecified |
|  | Food environment: marketing & advertising | Cost of resources | Majowicz et al., 2016 (4) | Unspecified |
|  | Food environment: marketing & advertising | Learning environment | Friel et al., 2017 (15) | Negative |
|  | Food environment: marketing & advertising | Healthy diet | Majowicz et al., 2016 (4) | Unspecified |
|  | Food environment: marketing & advertising | Alcohol & drug consumption | Holder and Blose, 1987 (25) | Positive |
|  | Food environment: marketing & advertising | Health-positive attitudes, beliefs, and preferences | Friel et al., 2017 (15); Zhang et al., 2014 (29) | Negative |
|  | Food environment: marketing & advertising | Health-positive knowledge & skills | Majowicz et al., 2016 (4) | Unspecified |
|  | Food environment: marketing & advertising | Social capital | Majowicz et al., 2016 (4) | Unspecified |
|  | Food environment: marketing & advertising | Health-positive childhood social influences | Majowicz et al., 2016 (4) | Unspecified |
|  | Food environment: marketing & advertising | Health-positive cultural and social norms | Majowicz et al., 2016 (4) | Unspecified |
|  | Physical activity environment: availability | Health-positive tendencies & habits | Blok et al., 2018 (30) | Unspecified |
|  | Physical activity environment: availability | Physical activity | Cavill et al., 2020 (7) | Unspecified |
|  | Physical activity environment: availability | Health-positive cultural and social norms | Cavill et al., 2020 (7) | Unspecified |
|  | Physical activity environment: accessibility | Cost of resources | Yang et al., 2015 (31) | Negative |
|  | Physical activity environment: accessibility | Physical activity | Cavill et al., 2020 (7) | Positive |
|  | Physical activity environment: accessibility | Health-positive tendencies & habits | Blok et al., 2018 (30) | Positive |
|  | Physical activity environment: accessibility | Health-positive attitudes, beliefs, and preferences | Blok et al., 2018 (30) | Positive |
|  | General physical environment: availability | General health | Majowicz et al., 2016 (4) | Unspecified |
|  | General physical environment: availability | Short-term & infectious disease | Majowicz et al., 2016 (4) | Unspecified |
|  | General physical environment: availability | Food environment: availability | Majowicz et al., 2016 (4) | Unspecified |
|  | General physical environment: availability | General physical environment: availability | Mills et al., 2023 (35); Cavill et al., 2020 (7) | Positive |
|  | General physical environment: availability | General physical environment: marketing & advertising | Mills et al., 2023 (35) | Positive |
|  | General physical environment: availability | Built environment | Cavill et al., 2020 (7) | Positive |
|  | General physical environment: availability | Financial strain | Majowicz et al., 2016 (4) | Unspecified |
|  | General physical environment: availability | Environmental hazards & pollutants | Majowicz et al., 2016 (4) | Unspecified |
|  | General physical environment: availability | Healthy diet | Majowicz et al., 2016 (4) | Unspecified |
|  | General physical environment: availability | Physical activity | Cavill et al., 2020 (7) | Unspecified |
|  | General physical environment: availability | Smoking | Mills et al., 2023 (35) | Negative |
|  | General physical environment: availability | Health-positive attitudes, beliefs, and preferences | Mills et al., 2023 (35) | Positive |
|  | General physical environment: availability | Health-positive cultural and social norms | Mills et al., 2023 (35) | Positive |
|  | General physical environment: accessibility | Food environment: accessibility | Friel et al., 2017 (15) | Positive |
|  | General physical environment: accessibility | Physical activity environment: accessibility | Blok et al., 2018 (30) | Positive |
|  | General physical environment: accessibility | Physical activity | Yang et al., 2011 (32) | Negative |
|  | General physical environment: accessibility | Bandwidth & time | Friel et al., 2017 (15); Sawyer et al., 2021 (16) | Positive |
|  | General physical environment: accessibility | Citizen engagement | Reumers et al., 2022 (5) | Positive |
|  | General physical environment: marketing & advertising | General physical environment: availability | Mills et al., 2023 (35) | Positive |
|  | General physical environment: marketing & advertising | Cost of resources | Mills et al., 2023 (35) | Positive |
|  | General physical environment: marketing & advertising | Health-positive tendencies & habits | Mills et al., 2023 (35) | Positive |
|  | Built environment | General health | Joffe, 2007 (1); Majowicz et al., 2016 (4) | Unspecified |
|  | Built environment | Mental health | Yang et al., 2019 (33) | Positive |
|  | Built environment | Obesity | Majowicz et al., 2016 (4) | Unspecified |
|  | Built environment | Short-term & infectious disease | Majowicz et al., 2016 (4) | Unspecified |
|  | Built environment | Treatment | Sturmberg et al., 2017 (18) | Unspecified |
|  | Built environment | Food environment: general | Friel et al., 2017 (15) | Positive |
|  | Built environment | Food environment: availability | Friel et al., 2017 (15); Majowicz et al., 2016 (4) | Positive |
|  | Built environment | General physical environment: availability | Cavill et al., 2020 (7) | Positive |
|  | Built environment | General physical environment: accessibility | Friel et al., 2017 (15) | Positive |
|  | Built environment | Built environment | Majowicz et al., 2016 (4); Cavill et al., 2020 (7) | Positive |
|  | Built environment | Natural environment | Majowicz et al., 2016 (4) | Unspecified |
|  | Built environment | Financial strain | Majowicz et al., 2016 (4) | Unspecified |
|  | Built environment | Cost of resources | Yang et al., 2015 (31) | Negative |
|  | Built environment | Environmental hazards & pollutants | Majowicz et al., 2016 (4); Cavill et al., 2020 (7) | Negative |
|  | Built environment | Neighborhood population | Brittin et al., 2015 (21); Friel et al., 2017 (15) | Positive |
|  | Built environment | Household demographics | Majowicz et al., 2016 (4) | Unspecified |
|  | Built environment | Housing | Friel et al., 2017 (15) | Positive |
|  | Built environment | Healthy diet | Majowicz et al., 2016 (4) | Unspecified |
|  | Built environment | Physical activity | Orr et al., 2016 (27); Yang et al., 2019 (33); Cavill et al., 2020 (7) | Positive |
|  | Built environment | Other behaviors | Joffe, 2007 (1) | Unspecified |
|  | Built environment | Health-positive attitudes, beliefs, and preferences | Friel et al., 2017 (15); Yang et al., 2011 (32); Yang et al., 2019 (33) | Positive |
|  | Built environment | Health-positive knowledge & skills | Friel et al., 2017 (15) | Positive |
|  | Built environment | Social cohesion | Majowicz et al., 2016 (4) | Unspecified |
|  | Built environment | Social capital | Majowicz et al., 2016 (4) | Unspecified |
|  | Built environment | Neo-liberal macroeconomic policies | Majowicz et al., 2016 (4) | Unspecified |
|  | Built environment | Health-positive cultural and social norms | Majowicz et al., 2016 (4); Cavill et al., 2020 (7) | Positive |
|  | Natural environment | General health | Weiler et al., 2015 (9) | Unspecified |
|  | Natural environment | Obesity | Majowicz et al., 2016 (4) | Unspecified |
|  | Natural environment | Short-term & infectious disease | Majowicz et al., 2016 (4) | Unspecified |
|  | Natural environment | Food environment: availability | Majowicz et al., 2016 (4) | Unspecified |
|  | Natural environment | General physical environment: availability | Majowicz et al., 2016 (4) | Unspecified |
|  | Natural environment | Natural environment | Majowicz et al., 2016 (4) | Unspecified |
|  | Natural environment | Financial strain | Majowicz et al., 2016 (4) | Unspecified |
|  | Natural environment | Environmental hazards & pollutants | Majowicz et al., 2016 (4) | Unspecified |
|  | Natural environment | Healthy diet | Majowicz et al., 2016 (4) | Unspecified |
|  | Natural environment | Socioeconomic position: general | Majowicz et al., 2016 (4) | Unspecified |
|  | Financial strain | General health | Joffe, 2007 (1) | Negative |
|  | Financial strain | Mental health | Yang et al., 2019 (33); Broomhead et al., 2021 (12) | Negative |
|  | Financial strain | Stress | Mills et al., 2023 (35); Reumers et al., 2022 (5) | Positive |
|  | Financial strain | Obesity | Majowicz et al., 2016 (4) | Unspecified |
|  | Financial strain | Food environment: accessibility | Sawyer et al., 2021 (16) | Negative |
|  | Financial strain | Food environment: perceived accessibility | Sawyer et al., 2021 (16) | Negative |
|  | Financial strain | Financial strain | Sawyer et al., 2021 (16); Broomhead et al., 2021 (12) | Positive |
|  | Financial strain | Cost of resources | Sawyer et al., 2021 (16); Reumers et al., 2022 (5); Salvo et al., 2022 (28) | Positive |
|  | Financial strain | Housing | Mills et al., 2023 (35) | Negative |
|  | Financial strain | Healthy diet | Majowicz et al., 2016 (4); Sawyer et al., 2021 (16); Broomhead et al., 2021 (12) | Negative |
|  | Financial strain | Bandwidth & time | Mills et al., 2023 (35) | Negative |
|  | Financial strain | Health-positive tendencies & habits | Sawyer et al., 2021 (16) | Negative |
|  | Financial strain | Health-positive attitudes, beliefs, and preferences | Sawyer et al., 2021 (16); Reumers et al., 2022 (5) | Negative |
|  | Financial strain | Health-positive knowledge & skills | Sawyer et al., 2021 (16) | Negative |
|  | Financial strain | Health-positive cultural and social norms | Sawyer et al., 2021 (16) | Positive |
|  | Financial strain | Stigma | Sawyer et al., 2021 (16) | Positive |
|  | Cost of resources | Stress | Reumers et al., 2022 (5) | Positive |
|  | Cost of resources | Obesity | Majowicz et al., 2016 (4) | Unspecified |
|  | Cost of resources | Health markers | Reumers et al., 2022 (5) | Negative |
|  | Cost of resources | Food environment: general | Majowicz et al., 2016 (4) | Unspecified |
|  | Cost of resources | Food environment: availability | Auchincloss et al., 2011 (23); Majowicz et al., 2016 (4) | Unspecified |
|  | Cost of resources | Food environment: perceived accessibility | Sawyer et al., 2021 (16) | Negative |
|  | Cost of resources | Financial strain | Majowicz et al., 2016 (4); Sawyer et al., 2021 (16); Reumers et al., 2022 (5) | Positive |
|  | Cost of resources | Cost of resources | Friel et al., 2017 (15); Sawyer et al., 2021 (16); Yang et al., 2015 (31); Reumers et al., 2022 (5); Salvo et al., 2022 (28) | Positive |
|  | Cost of resources | Risk behaviors: general | Reumers et al., 2022 (5) | Negative |
|  | Cost of resources | Healthy diet | Auchincloss et al., 2011 (23); Blok et al., 2015 (24); Friel et al., 2017 (15);Joffe, 2007 (1); Majowicz et al., 2016 (4); Zhang et al., 2014 (29); Li et al., 2018 (13) | Conflicting |
|  | Cost of resources | Alcohol & drug consumption | Holder and Blose, 1987 (25) | Negative |
|  | Cost of resources | Smoking | Mills et al., 2023 (35) | Negative |
|  | Cost of resources | Care-seeking behavior | Reumers et al., 2022 (5) | Negative |
|  | Cost of resources | Health-positive tendencies & habits | Blok et al., 2018 (30); Friel et al., 2017 (15); Yang et al., 2015 (31) | Negative |
|  | Cost of resources | Health-positive attitudes, beliefs, and preferences | Blok et al., 2018 (30); Yang et al., 2019 (33); Salvo et al., 2022 (28) | Negative |
|  | Cost of resources | Income | Friel et al., 2017 (15) | Positive |
|  | Cost of resources | Education | Joffe, 2007 (1)(1) | Unspecified |
|  | Cost of resources | Citizen engagement | Reumers et al., 2022 (5) | Negative |
|  | Working conditions | General health | Weiler et al., 2015 (9) | Unspecified |
|  | Working conditions | Short-term & infectious disease | Joffe, 2007 (1) | Negative |
|  | Working conditions | General physical environment: availability | Cavill et al., 2020 (7) | Positive |
|  | Working conditions | General physical environment: accessibility | Friel et al., 2017 (15) | Positive |
|  | Working conditions | Working conditions | Friel et al., 2017 (15); Joffe, 2007 (1) | Positive |
|  | Working conditions | Environmental hazards & pollutants | Joffe, 2007 (1) | Negative |
|  | Working conditions | Housing | Friel et al., 2017 (15) | Positive |
|  | Working conditions | Healthy diet | Joffe, 2007 (1) | Positive |
|  | Working conditions | Physical activity | Cavill et al., 2020 (7) | Unspecified |
|  | Working conditions | Income | Friel et al., 2017 (15) | Positive |
|  | Working conditions | Education | Joffe, 2007 (1) | Positive |
|  | Violence | Mental health | Occhipinti et al., 2021 (11); Mooney et al., 2022 (10); Broomhead et al., 2021 (12) | Negative |
|  | Violence | Stress | Mooney et al., 2022 (10) | Unspecified |
|  | Violence | Housing | Occhipinti et al., 2021 (11) | Unspecified |
|  | Violence | Health markers | Broomhead et al., 2021 (12) | Positive |
|  | Violence | Violence | Mooney et al., 2022 (10) | Positive |
|  | Violence | Household demographics | Mooney et al., 2022 (10) | Unspecified |
|  | Violence | Alcohol & drug consumption | Mooney et al., 2022 (10) | Unspecified |
|  | Violence | Health-positive intention & motivation | Broomhead et al., 2021 (12) | Positive |
|  | Violence | Health-positive childhood social influences | Occhipinti et al., 2021 (11) | Unspecified |
|  | Violence | Income | Mooney et al., 2022 (10) | Unspecified |
|  | Environmental hazards & pollutants | General health | Joffe, 2007 (1); Majowicz et al., 2016 (4); Neff et al., 2009 (22); Milstein et al., 2010 (19) | Negative |
|  | Environmental hazards & pollutants | Obesity | Majowicz et al., 2016 (4) | Unspecified |
|  | Environmental hazards & pollutants | Short-term & infectious disease | Majowicz et al., 2016 (4); Milstein et al., 2010 (19) | Positive |
|  | Environmental hazards & pollutants | Health markers | Majowicz et al., 2016 (4) | Unspecified |
|  | Environmental hazards & pollutants | General physical environment: availability | Majowicz et al., 2016 (4) | Unspecified |
|  | Environmental hazards & pollutants | Natural environment | Majowicz et al., 2016 (4) | Unspecified |
|  | Environmental hazards & pollutants | Financial strain | Majowicz et al., 2016 (4) | Unspecified |
|  | Environmental hazards & pollutants | Environmental hazards & pollutants | Joffe, 2007 (1); Majowicz et al., 2016 (4) | Positive |
|  | Environmental hazards & pollutants | Risk behaviors: general | Milstein et al., 2010 (19) | Positive |
|  | Environmental hazards & pollutants | Physical activity | Cavill et al., 2020 (7) | Negative |
|  | Environmental hazards & pollutants | Health-enhancing biological factors | Majowicz et al., 2016 (4) | Unspecified |
|  | Environmental hazards & pollutants | Health-positive tendencies & habits | Blok et al., 2018 (30) | Negative |
|  | Environmental hazards & pollutants | Health-positive attitudes, beliefs, and preferences | Yang et al., 2011 (32) | Negative |
|  | Environmental hazards & pollutants | Social capital | Neff et al., 2009 (22); Mills et al., 2023 (35) | Negative |
|  | Environmental hazards & pollutants | Health-positive cultural and social norms | Mills et al., 2023 (35) | Negative |
|  | Neighborhood income | Food environment: availability | Orr et al., 2014 (26); Orr et al., 2016 (27) | Positive |
|  | Neighborhood income | Built environment | Orr et al., 2016 (27) | Positive |
|  | Neighborhood income | Learning environment | Orr et al., 2014 (26); Orr et al., 2016 (27) | Positive |
|  | Neighborhood population | Obesity | Majowicz et al., 2016 (4) | Unspecified |
|  | Neighborhood population | Short-term & infectious disease | Majowicz et al., 2016 (4) | Unspecified |
|  | Neighborhood population | Food environment: availability | Sawyer et al., 2021 (16) | Positive |
|  | Neighborhood population | Violence | Mooney et al., 2022 (10) | Unspecified |
|  | Neighborhood population | Neighborhood population | Brittin et al., 2015 (21);Mahamoud et al., 2013 (8) | Conflicting |
|  | Neighborhood population | Housing | Brittin et al., 2015 (21) | Negative |
|  | Neighborhood population | Income | Brittin et al., 2015 (21) | Conflicting |
|  | Neighborhood population | Market-focused economic environment | Friel et al., 2017 (15) | Positive |
|  | Household demographics | General health | Lymer and Brown, 2012 (2); Majowicz et al., 2016 (4); Weiler et al., 2015 (9) | Unspecified |
|  | Household demographics | Mental health | Mooney et al., 2022 (10) | Unspecified |
|  | Household demographics | Stress | Mooney et al., 2022 (10) | Unspecified |
|  | Household demographics | Obesity | Lymer and Brown, 2012 (2); Majowicz et al., 2016 (4) | Unspecified |
|  | Household demographics | Health system attributes: general | Lymer and Brown, 2012 (2) | Unspecified |
|  | Household demographics | Access to healthcare | Majowicz et al., 2016 (4) | Unspecified |
|  | Household demographics | Financial strain | Majowicz et al., 2016 (4) | Unspecified |
|  | Household demographics | Cost of resources | Joffe, 2007 (1) | Unspecified |
|  | Household demographics | Working conditions | Joffe, 2007 (1) | Unspecified |
|  | Household demographics | Violence | Mooney et al., 2022 (10) | Unspecified |
|  | Household demographics | Household demographics | Joffe, 2007 (1) | Unspecified |
|  | Household demographics | Healthy diet | Majowicz et al., 2016 (4) | Unspecified |
|  | Household demographics | Physical activity | Lymer and Brown, 2012 (2); Cavill et al., 2020 (7) | Positive |
|  | Household demographics | Alcohol & drug consumption | Lymer and Brown, 2012 (2); Mooney et al., 2022 (10) | Unspecified |
|  | Household demographics | Smoking | Lymer and Brown, 2012 (2) | Unspecified |
|  | Household demographics | Bandwidth & time | Cavill et al., 2020 (7) | Negative |
|  | Household demographics | Health-positive knowledge & skills | Joffe, 2007 (1) | Unspecified |
|  | Household demographics | Health-positive intention & motivation | Rahmani et al., 2021 (34) | Unspecified |
|  | Household demographics | Social capital | Majowicz et al., 2016 (4) | Unspecified |
|  | Household demographics | Health-positive childhood social influences | Majowicz et al., 2016 (4) | Unspecified |
|  | Household demographics | Income | Mooney et al., 2022 (10); Cavill et al., 2020 (7) | Negative |
|  | Household demographics | Regulatory governance | Majowicz et al., 2016 (4) | Unspecified |
|  | Learning environment | Health markers | Broomhead et al., 2021 (12) | Unspecified |
|  | Learning environment | Other behaviors | Orr et al., 2014 (26); Orr et al., 2016 (27) | Unspecified |
|  | Learning environment | Health-positive knowledge & skills | Friel et al., 2017 (15); Reumers et al., 2022 (5) | Positive |
|  | Learning environment | Education | Orr et al., 2014 (26); Orr et al., 2016 (27) | Positive |
|  | Housing | General health | Joffe, 2007 (1) | Positive |
|  | Housing | Mental health | Occhipinti et al., 2021 (11); Broomhead et al., 2021 (12) | Positive |
|  | Housing | Stress | Mills et al., 2023 (35) | Negative |
|  | Housing | Chronic disease | Mahamoud et al., 2013 (8) | Unspecified |
|  | Housing | Treatment | Sturmberg et al., 2017 (18) | Unspecified |
|  | Housing | Food environment: accessibility | Friel et al., 2017 (15) | Positive |
|  | Housing | Financial strain | Broomhead et al., 2021 (12) | Negative |
|  | Housing | Neighborhood population | Brittin et al., 2015 (21) | Positive |
|  | Non-modifiable risk factors | Chronic disease | Homa et al., 2015 (17) | Unspecified |
| Behavior | Risk behaviors: general | General health | Ansari et al., 2003 (6); Milstein et al., 2010 (19); Reumers et al., 2022 (5) | Negative |
|  | Risk behaviors: general | Chronic disease | Homa et al., 2015 (17);Mahamoud et al., 2013 (8); Milstein et al., 2010 (19) | Positive |
|  | Risk behaviors: general | Short-term & infectious disease | Milstein et al., 2010 (19) | Positive |
|  | Risk behaviors: general | Cost of resources | Reumers et al., 2022 (5) | Positive |
|  | Risk behaviors: general | Socioeconomic position: general | Ansari et al., 2003 (6) | Unspecified |
|  | Healthy diet | General health | Joffe, 2007 (1); Majowicz et al., 2016 (4); Neff et al., 2009 (22); Weiler et al., 2015 (9) | Positive |
|  | Healthy diet | Stress | Sawyer et al., 2021 (16) | Negative |
|  | Healthy diet | Chronic disease | Orr et al., 2014 (26); Joffe, 2007 (1); Broomhead et al., 2021 (12) | Negative |
|  | Healthy diet | Obesity | Majowicz et al., 2016 (4) | Unspecified |
|  | Healthy diet | Short-term & infectious disease | Joffe, 2007 (1) | Negative |
|  | Healthy diet | Health markers | Majowicz et al., 2016 (4); Orr et al., 2014 (26); Orr et al., 2016 (27) | Positive |
|  | Healthy diet | Treatment | Sturmberg et al., 2017 (18) | Unspecified |
|  | Healthy diet | Food environment: general | Neff et al., 2009 (22) | Unspecified |
|  | Healthy diet | Food environment: availability | Auchincloss et al., 2011 (23); Blok et al., 2015 (24); Majowicz et al., 2016 (4); Salvo et al., 2022 (28) | Conflicting |
|  | Healthy diet | Financial strain | Majowicz et al., 2016 (4) | Unspecified |
|  | Healthy diet | Environmental hazards & pollutants | Majowicz et al., 2016 (4) | Unspecified |
|  | Healthy diet | Healthy diet | Joffe, 2007 (1); Majowicz et al., 2016 (4); Broomhead et al., 2021 (12) | Positive |
|  | Healthy diet | Health-enhancing biological factors | Majowicz et al., 2016 (4); Sawyer et al., 2021 (16) | Positive |
|  | Healthy diet | Health-positive intention & motivation | Rahmani et al., 2021 (34) | Positive |
|  | Healthy diet | Social capital | Rahmani et al., 2021 (34) | Unspecified |
|  | Healthy diet | Health-positive childhood social influences | Majowicz et al., 2016 (4) | Unspecified |
|  | Physical activity | General health | Lymer and Brown, 2012 (2); Cavill et al., 2020 (7) | Positive |
|  | Physical activity | Mental health | Yang et al., 2019 (33) | Positive |
|  | Physical activity | Obesity | Lymer and Brown, 2012 (2) | Unspecified |
|  | Physical activity | Health markers | Orr et al., 2016 (27) | Positive |
|  | Physical activity | Financial strain | Yang et al., 2019 (33) | Unspecified |
|  | Physical activity | Physical activity | Lymer and Brown, 2012 (2); Yang et al., 2019 (33); Cavill et al., 2020 (7) | Positive |
|  | Physical activity | Alcohol & drug consumption | Lymer and Brown, 2012 (2) | Unspecified |
|  | Physical activity | Smoking | Lymer and Brown, 2012 (2) | Unspecified |
|  | Physical activity | Health-enhancing biological factors | Wittenborn et al., 2015 (3) | Positive |
|  | Physical activity | Health-positive tendencies & habits | Cavill et al., 2020 (7) | Positive |
|  | Physical activity | Health-positive attitudes, beliefs, and preferences | Yang et al., 2011 (32); Yang et al., 2019 (33); Cavill et al., 2020 (7) | Conflicting |
|  | Physical activity | Health-positive knowledge & skills | Cavill et al., 2020 (7) | Positive |
|  | Physical activity | Social capital | Yang et al., 2019 (33) | Unspecified |
|  | Alcohol & drug consumption | General health | Lymer and Brown, 2012 (2) | Unspecified |
|  | Alcohol & drug consumption | Mental health | Occhipinti et al., 2021 (11); Mooney et al., 2022 (10) | Unspecified |
|  | Alcohol & drug consumption | Stress | Mooney et al., 2022 (10) | Unspecified |
|  | Alcohol & drug consumption | Violence | Occhipinti et al., 2021 (11); Mooney et al., 2022 (10) | Unspecified |
|  | Alcohol & drug consumption | Household demographics | Mooney et al., 2022 (10) | Unspecified |
|  | Alcohol & drug consumption | Physical activity | Lymer and Brown, 2012 (2) | Unspecified |
|  | Alcohol & drug consumption | Alcohol & drug consumption | Lymer and Brown, 2012 (2) | Unspecified |
|  | Alcohol & drug consumption | Smoking | Lymer and Brown, 2012 (2) | Unspecified |
|  | Alcohol & drug consumption | Health-positive childhood social influences | Occhipinti et al., 2021 (11) | Unspecified |
|  | Alcohol & drug consumption | Income | Mooney et al., 2022 (10) | Unspecified |
|  | Alcohol & drug consumption | Employment | Occhipinti et al., 2021 (11) | Unspecified |
|  | Smoking | General health | Lymer and Brown, 2012 (2) | Unspecified |
|  | Smoking | Financial strain | Mills et al., 2023 (35) | Positive |
|  | Smoking | Physical activity | Lymer and Brown, 2012 (2) | Unspecified |
|  | Smoking | Alcohol & drug consumption | Lymer and Brown, 2012 (2) | Unspecified |
|  | Smoking | Health-positive tendencies & habits | Mills et al., 2023 (35) | Negative |
|  | Smoking | Health-positive cultural and social norms | Mills et al., 2023 (35) | Negative |
|  | Smoking | Structural discrimination | Mills et al., 2023 (35) | Positive |
|  | Sleep | Health-enhancing biological factors | Wittenborn et al., 2015 (3) | Positive |
|  | Care-seeking behavior | General health | Reumers et al., 2022 (5) | Positive |
|  | Care-seeking behavior | Mental health | Occhipinti et al., 2021 (11) | Unspecified |
|  | Care-seeking behavior | Obesity | Majowicz et al., 2016 (4) | Unspecified |
|  | Care-seeking behavior | Health markers | Broomhead et al., 2021 (12) | Positive |
|  | Care-seeking behavior | Healthcare costs | Milstein et al., 2010 (19); Reumers et al., 2022 (5) | Positive |
|  | Care-seeking behavior | Care-seeking behavior | Reumers et al., 2022 (5) | Positive |
|  | Care-seeking behavior | Risk behaviors: general | Homa et al., 2015 (17) | Positive |
|  | Care-seeking behavior | Health-enhancing biological factors | Majowicz et al., 2016 (4) | Unspecified |
|  | Care-seeking behavior | Health-positive tendencies & habits | Homa et al., 2015 (17) | Positive |
|  | Suicidal behavior | Care-seeking behavior | Occhipinti et al., 2021 (11) | Unspecified |
|  | Other behaviors | General health | Wittenborn et al., 2015 (3) | Unspecified |
|  | Other behaviors | Chronic disease | Broomhead et al., 2021 (12) | Unspecified |
|  | Other behaviors | Health-enhancing biological factors | Sawyer et al., 2021 (16); Wittenborn et al., 2015 (3) | Unspecified |
|  | Other behaviors | Social capital | Wittenborn et al., 2015 (3) | Unspecified |
|  | Other behaviors | Socioeconomic position: general | Wittenborn et al., 2015 (3) | Unspecified |
|  | Other behaviors | Education | Orr et al., 2014 (26); Orr et al., 2016 (27) | Unspecified |
| Biological factors | Age | General health | Lymer and Brown, 2012 (2); Yang et al., 2019 (33) | Unspecified |
|  | Age | Mental health | Yang et al., 2019 (33); Mooney et al., 2022 (10) | Unspecified |
|  | Age | Stress | Mooney et al., 2022 (10) | Unspecified |
|  | Age | Chronic disease | Cavill et al., 2020 (7) | Positive |
|  | Age | Obesity | Lymer and Brown, 2012 (2); Majowicz et al., 2016 (4) | Unspecified |
|  | Age | Health system attributes: general | Lymer and Brown, 2012 (2) | Unspecified |
|  | Age | Healthcare costs | Lymer and Brown, 2012 (2) | Unspecified |
|  | Age | Violence | Mooney et al., 2022 (10) | Unspecified |
|  | Age | Household demographics | Mooney et al., 2022 (10) | Unspecified |
|  | Age | Healthy diet | Zhang et al., 2014 (29); Li et al., 2018 (13) | Positive |
|  | Age | Physical activity | Lymer and Brown, 2012 (2) | Unspecified |
|  | Age | Alcohol & drug consumption | Lymer and Brown, 2012 (2) | Unspecified |
|  | Age | Smoking | Lymer and Brown, 2012 (2) | Unspecified |
|  | Age | Care-seeking behavior | Lymer and Brown, 2012 (2) | Unspecified |
|  | Age | Attainable health | Yang et al., 2011 (32) | Negative |
|  | Age | Health-enhancing biological factors | Majowicz et al., 2016 (4) | Unspecified |
|  | Age | Health-positive tendencies & habits | Blok et al., 2018 (30) | Negative |
|  | Age | Health-positive attitudes, beliefs, and preferences | Li et al., 2018 (13) | Positive |
|  | Age | Social capital | Majowicz et al., 2016 (4) | Unspecified |
|  | Age | Income | Mooney et al., 2022 (10) | Unspecified |
|  | Age | Employment | Yang et al., 2019 (33) | Unspecified |
|  | Attainable health | Care-seeking behavior | Homa et al., 2015 (17) | Unspecified |
|  | Health-enhancing biological factors | Stress | Wittenborn et al., 2015 (3); Crielaard et al., 2021 (14) | Negative |
|  | Health-enhancing biological factors | Chronic disease | Sturmberg et al., 2017 (18) | Unspecified |
|  | Health-enhancing biological factors | Health markers | Majowicz et al., 2016 (4); Sawyer et al., 2021 (16) | Positive |
|  | Health-enhancing biological factors | Treatment | Sturmberg et al., 2017 (18) | Unspecified |
|  | Health-enhancing biological factors | Built environment | Sturmberg et al., 2017 (18) | Unspecified |
|  | Health-enhancing biological factors | Household demographics | Joffe, 2007 (1) | Unspecified |
|  | Health-enhancing biological factors | Healthy diet | Sturmberg et al., 2017 (18) | Unspecified |
|  | Health-enhancing biological factors | Sleep | Wittenborn et al., 2015 (3) | Unspecified |
|  | Health-enhancing biological factors | Other behaviors | Wittenborn et al., 2015 (3) | Unspecified |
|  | Health-enhancing biological factors | Health-enhancing biological factors | Majowicz et al., 2016 (4); Sturmberg et al., 2017 (18); Wittenborn et al., 2015 (3) | Positive |
|  | Health-enhancing biological factors | Social capital | Sturmberg et al., 2017 (18); Wittenborn et al., 2015 (3) | Positive |
|  | Health-enhancing biological factors | Health-positive childhood social influences | Majowicz et al., 2016 (4) | Unspecified |
|  | Health-enhancing biological factors | Socioeconomic position: general | Wittenborn et al., 2015 (3) | Positive |
|  | Health-enhancing biological factors | Employment | Sturmberg et al., 2017 (18) | Unspecified |
|  | Health-enhancing biological factors | Education | Sturmberg et al., 2017 (18) | Unspecified |
| Individual-level determinants of behavior change | Health-positive psychosocial factors: general | General health | Ansari et al., 2003 (6) | Unspecified |
|  | Health-positive psychosocial factors: general | Health system attributes: general | Ansari et al., 2003 (6) | Unspecified |
|  | Health-positive psychosocial factors: general | Risk behaviors: general | Ansari et al., 2003 (6) | Unspecified |
|  | Health-positive psychosocial factors: general | Socioeconomic position: general | Ansari et al., 2003 (6) | Unspecified |
|  | Health-positive psychosocial factors: general | Health-positive cultural and social norms | Ansari et al., 2003 (6) | Unspecified |
|  | Bandwidth & time | Stress | Friel et al., 2017 (15) | Negative |
|  | Bandwidth & time | Obesity | Majowicz et al., 2016 (4) | Unspecified |
|  | Bandwidth & time | Food environment: availability | Majowicz et al., 2016 (4); Salvo et al., 2022 (28) | Unspecified |
|  | Bandwidth & time | Food environment: accessibility | Sawyer et al., 2021 (16) | Positive |
|  | Bandwidth & time | Physical activity environment: accessibility | Cavill et al., 2020 (7) | Negative |
|  | Bandwidth & time | Financial strain | Majowicz et al., 2016 (4) | Unspecified |
|  | Bandwidth & time | Cost of resources | Sawyer et al., 2021 (16) | Negative |
|  | Bandwidth & time | Healthy diet | Majowicz et al., 2016 (4); Rahmani et al., 2021 (34); Salvo et al., 2022 (28) | Unspecified |
|  | Bandwidth & time | Smoking | Mills et al., 2023 (35) | Negative |
|  | Bandwidth & time | Bandwidth & time | Friel et al., 2017 (15) | Positive |
|  | Bandwidth & time | Health-positive tendencies & habits | Sawyer et al., 2021 (16); Crielaard et al., 2021 (14) | Positive |
|  | Bandwidth & time | Health-positive attitudes, beliefs, and preferences | Cavill et al., 2020 (7) | Positive |
|  | Bandwidth & time | Health-positive knowledge & skills | Friel et al., 2017 (15); Majowicz et al., 2016 (4) | Positive |
|  | Bandwidth & time | Employment | Reumers et al., 2022 (5) | Positive |
|  | Bandwidth & time | Education | Reumers et al., 2022 (5) | Positive |
|  | Health-positive tendencies & habits | Stress | Crielaard et al., 2021 (14) | Unspecified |
|  | Health-positive tendencies & habits | Food environment: availability | Friel et al., 2017 (15); Sawyer et al., 2021 (16) | Positive |
|  | Health-positive tendencies & habits | Food environment: accessibility | Friel et al., 2017 (15) | Positive |
|  | Health-positive tendencies & habits | Food environment: marketing & advertising | Sawyer et al., 2021 (16) | Positive |
|  | Health-positive tendencies & habits | Financial strain | Sawyer et al., 2021 (16) | Negative |
|  | Health-positive tendencies & habits | Cost of resources | Yang et al., 2015 (31) | Conflicting |
|  | Health-positive tendencies & habits | Healthy diet | Auchincloss et al., 2011 (23) | Positive |
|  | Health-positive tendencies & habits | Physical activity | Cavill et al., 2020 (7) | Positive |
|  | Health-positive tendencies & habits | Smoking | Mills et al., 2023 (35) | Negative |
|  | Health-positive tendencies & habits | Care-seeking behavior | Homa et al., 2015 (17) | Unspecified |
|  | Health-positive tendencies & habits | Health-enhancing biological factors | Crielaard et al., 2021 (14) | Unspecified |
|  | Health-positive tendencies & habits | Health-positive tendencies & habits | Blok et al., 2018 (30); Sawyer et al., 2021 (16) | Positive |
|  | Health-positive tendencies & habits | Health-positive attitudes, beliefs, and preferences | Sawyer et al., 2021 (16); Cavill et al., 2020 (7) | Positive |
|  | Health-positive tendencies & habits | Health-positive knowledge & skills | Sawyer et al., 2021 (16); Crielaard et al., 2021 (14) | Conflicting |
|  | Health-positive tendencies & habits | Health-positive intention & motivation | Sawyer et al., 2021 (16) | Positive |
|  | Health-positive tendencies & habits | Health-positive cultural and social norms | Friel et al., 2017 (15); Sawyer et al., 2021 (16) | Positive |
|  | Health-positive attitudes, beliefs, and preferences | General health | Reumers et al., 2022 (5) | Positive |
|  | Health-positive attitudes, beliefs, and preferences | Food environment: availability | Friel et al., 2017 (15); Sawyer et al., 2021 (16) | Positive |
|  | Health-positive attitudes, beliefs, and preferences | Food environment: perceived accessibility | Sawyer et al., 2021 (16) | Positive |
|  | Health-positive attitudes, beliefs, and preferences | Physical activity environment: availability | Blok et al., 2018 (30) | Conflicting |
|  | Health-positive attitudes, beliefs, and preferences | General physical environment: availability | Mills et al., 2023 (35) | Positive |
|  | Health-positive attitudes, beliefs, and preferences | Healthy diet | Auchincloss et al., 2011 (23); Blok et al., 2015 (24); Zhang et al., 2014 (29); Li et al., 2018 (13); Salvo et al., 2022 (28) | Positive |
|  | Health-positive attitudes, beliefs, and preferences | Physical activity | Yang et al., 2011 (32); Yang et al., 2019 (33); Cavill et al., 2020 (7) | Positive |
|  | Health-positive attitudes, beliefs, and preferences | Smoking | Mills et al., 2023 (35) | Negative |
|  | Health-positive attitudes, beliefs, and preferences | Care-seeking behavior | Reumers et al., 2022 (5) | Positive |
|  | Health-positive attitudes, beliefs, and preferences | Bandwidth & time | Friel et al., 2017 (15) | Positive |
|  | Health-positive attitudes, beliefs, and preferences | Health-positive tendencies & habits | Yang et al., 2015 (31); Sawyer et al., 2021 (16); Cavill et al., 2020 (7) | Positive |
|  | Health-positive attitudes, beliefs, and preferences | Health-positive attitudes, beliefs, and preferences | Friel et al., 2017 (15); Yang et al., 2015 (31); Yang et al., 2019 (33); Zhang et al., 2014 (29); Li et al., 2018 (13); Mills et al., 2023 (35); Cavill et al., 2020 (7) | Positive |
|  | Health-positive attitudes, beliefs, and preferences | Social capital | Friel et al., 2017 (15); Reumers et al., 2022 (5) | Positive |
|  | Health-positive attitudes, beliefs, and preferences | Market-focused economic environment | Sawyer et al., 2021 (16) | Positive |
|  | Health-positive attitudes, beliefs, and preferences | Health-positive cultural and social norms | Friel et al., 2017 (15); Sawyer et al., 2021 (16) | Positive |
|  | Health-positive knowledge & skills | Stress | Reumers et al., 2022 (5); Crielaard et al., 2021 (14) | Negative |
|  | Health-positive knowledge & skills | Obesity | Majowicz et al., 2016 (4) | Unspecified |
|  | Health-positive knowledge & skills | Financial strain | Majowicz et al., 2016 (4); Sawyer et al., 2021 (16) | Negative |
|  | Health-positive knowledge & skills | Cost of resources | Reumers et al., 2022 (5) | Negative |
|  | Health-positive knowledge & skills | Working conditions | Joffe, 2007 (1) | Positive |
|  | Health-positive knowledge & skills | Risk behaviors: general | Reumers et al., 2022 (5) | Negative |
|  | Health-positive knowledge & skills | Healthy diet | Majowicz et al., 2016 (4) | Unspecified |
|  | Health-positive knowledge & skills | Care-seeking behavior | Reumers et al., 2022 (5) | Positive |
|  | Health-positive knowledge & skills | Health-positive tendencies & habits | Sawyer et al., 2021 (16); Crielaard et al., 2021 (14); Cavill et al., 2020 (7) | Positive |
|  | Health-positive knowledge & skills | Health-positive attitudes, beliefs, and preferences | Friel et al., 2017 (15); Sawyer et al., 2021 (16); Reumers et al., 2022 (5) | Positive |
|  | Health-positive knowledge & skills | Health-positive knowledge & skills | Friel et al., 2017 (15); Reumers et al., 2022 (5); Crielaard et al., 2021 (14) | Positive |
|  | Health-positive knowledge & skills | Health-positive intention & motivation | Sawyer et al., 2021 (16) | Positive |
|  | Health-positive knowledge & skills | Generous social policies | Friel et al., 2017 (15) | Positive |
|  | Health-positive knowledge & skills | Health-positive cultural and social norms | Friel et al., 2017 (15); Sawyer et al., 2021 (16) | Positive |
|  | Health-positive intention & motivation | Chronic disease | Broomhead et al., 2021 (12) | Positive |
|  | Health-positive intention & motivation | Healthy diet | Rahmani et al., 2021 (34) | Positive |
|  | Health-positive intention & motivation | Health-positive attitudes, beliefs, and preferences | Sawyer et al., 2021 (16) | Positive |
|  | Health-positive intention & motivation | Health-positive cultural and social norms | Sawyer et al., 2021 (16) | Positive |
|  | Sensitivity to social norms | Health-positive attitudes, beliefs, and preferences | Zhang et al., 2014 (29); Li et al., 2018 (13) | Positive |
| Social cohesion | Social cohesion | General health | Majowicz et al., 2016 (4) | Unspecified |
|  | Social cohesion | Obesity | Majowicz et al., 2016 (4) | Unspecified |
|  | Social cohesion | Built environment | Majowicz et al., 2016 (4) | Unspecified |
|  | Social cohesion | Financial strain | Majowicz et al., 2016 (4) | Unspecified |
|  | Social cohesion | Neighborhood population | Brittin et al., 2015 (21) | Positive |
|  | Social cohesion | Household demographics | Majowicz et al., 2016 (4) | Unspecified |
|  | Social cohesion | Housing | Mahamoud et al., 2013 (8) | Negative |
|  | Social cohesion | Health-positive tendencies & habits | Blok et al., 2018 (30) | Positive |
|  | Social cohesion | Social capital | Majowicz et al., 2016 (4) | Unspecified |
|  | Social cohesion | Income | Mahamoud et al., 2013 (8) | Negative |
|  | Social cohesion | Regulatory governance | Majowicz et al., 2016 (4) | Unspecified |
|  | Social cohesion | Health-positive cultural and social norms | Majowicz et al., 2016 (4) | Unspecified |
|  | Social capital | General health | Reumers et al., 2022 (5) | Positive |
|  | Social capital | Mental health | Yang et al., 2019 (33) | Unspecified |
|  | Social capital | Stress | Mills et al., 2023 (35) | Negative |
|  | Social capital | Chronic disease | Broomhead et al., 2021 (12) | Positive |
|  | Social capital | Obesity | Majowicz et al., 2016 (4) | Unspecified |
|  | Social capital | Health markers | Reumers et al., 2022 (5) | Positive |
|  | Social capital | Treatment | Sturmberg et al., 2017 (18) | Unspecified |
|  | Social capital | Food environment: general | Neff et al., 2009 (22) | Unspecified |
|  | Social capital | General physical environment: availability | Mills et al., 2023 (35) | Positive |
|  | Social capital | Built environment | Majowicz et al., 2016 (4) | Unspecified |
|  | Social capital | Financial strain | Majowicz et al., 2016 (4); Sawyer et al., 2021 (16) | Negative |
|  | Social capital | Environmental hazards & pollutants | Neff et al., 2009 (22); Mills et al., 2023 (35) | Negative |
|  | Social capital | Household demographics | Majowicz et al., 2016 (4) | Unspecified |
|  | Social capital | Healthy diet | Majowicz et al., 2016 (4); Neff et al., 2009 (22); Orr et al., 2014 (26) | Unspecified |
|  | Social capital | Health-enhancing biological factors | Wittenborn et al., 2015 (3) | Positive |
|  | Social capital | Health-positive tendencies & habits | Blok et al., 2018 (30); Cavill et al., 2020 (7) | Positive |
|  | Social capital | Health-positive attitudes, beliefs, and preferences | Friel et al., 2017 (15); Yang et al., 2015 (31); Yang et al., 2011 (32); Zhang et al., 2014 (29); Mills et al., 2023 (35); Cavill et al., 2020 (7) | Positive |
|  | Social capital | Health-positive knowledge & skills | Friel et al., 2017 (15); Majowicz et al., 2016 (4); Reumers et al., 2022 (5); Cavill et al., 2020 (7) | Positive |
|  | Social capital | Health-positive intention & motivation | Rahmani et al., 2021 (34) | Positive |
|  | Social capital | Social cohesion | Majowicz et al., 2016 (4) | Unspecified |
|  | Social capital | Social capital | Yang et al., 2015 (31) | Positive |
|  | Social capital | Income | Friel et al., 2017 (15) | Positive |
|  | Social capital | Health-positive cultural and social norms | Friel et al., 2017 (15); Sawyer et al., 2021 (16); Mills et al., 2023 (35) | Positive |
|  | Social capital | Citizen engagement | Reumers et al., 2022 (5) | Positive |
|  | Health-positive childhood social influences | Mental health | Occhipinti et al., 2021 (11) | Unspecified |
|  | Health-positive childhood social influences | Obesity | Majowicz et al., 2016 (4) | Unspecified |
|  | Health-positive childhood social influences | Health markers | Sawyer et al., 2021 (16) | Positive |
|  | Health-positive childhood social influences | Food environment: availability | Sawyer et al., 2021 (16) | Unspecified |
|  | Health-positive childhood social influences | Financial strain | Sawyer et al., 2021 (16) | Negative |
|  | Health-positive childhood social influences | Working conditions | Joffe, 2007 (1) | Positive |
|  | Health-positive childhood social influences | Household demographics | Majowicz et al., 2016 (4) | Unspecified |
|  | Health-positive childhood social influences | Housing | Occhipinti et al., 2021 (11) | Unspecified |
|  | Health-positive childhood social influences | Healthy diet | Majowicz et al., 2016 (4) | Unspecified |
|  | Health-positive childhood social influences | Physical activity | Cavill et al., 2020 (7) | Positive |
|  | Health-positive childhood social influences | Alcohol & drug consumption | Occhipinti et al., 2021 (11) | Unspecified |
|  | Health-positive childhood social influences | Other behaviors | Sawyer et al., 2021 (16) | Unspecified |
|  | Health-positive childhood social influences | Health-enhancing biological factors | Joffe, 2007 (1); Majowicz et al., 2016 (4); Wittenborn et al., 2015 (3) | Positive |
|  | Health-positive childhood social influences | Health-positive knowledge & skills | Crielaard et al., 2021 (14) | Positive |
|  | Health-positive childhood social influences | Social capital | Cavill et al., 2020 (7) | Positive |
|  | Health-positive childhood social influences | Health-positive childhood social influences | Majowicz et al., 2016 (4); Sawyer et al., 2021 (16) | Unspecified |
|  | Health-positive childhood social influences | Education | Joffe, 2007 (1) | Positive |
|  | Health-positive childhood social influences | Health-positive cultural and social norms | Sawyer et al., 2021 (16) | Positive |
| Social stratification | Socioeconomic position: general | General health | Ansari et al., 2003 (6) | Positive |
|  | Socioeconomic position: general | Chronic disease | Milstein et al., 2010 (19); Broomhead et al., 2021 (12) | Negative |
|  | Socioeconomic position: general | Obesity | Majowicz et al., 2016 (4) | Unspecified |
|  | Socioeconomic position: general | Health markers | Majowicz et al., 2016 (4) | Unspecified |
|  | Socioeconomic position: general | Health system attributes: general | Majowicz et al., 2016 (4) | Unspecified |
|  | Socioeconomic position: general | Food environment: general | Neff et al., 2009 (22) | Unspecified |
|  | Socioeconomic position: general | Natural environment | Majowicz et al., 2016 (4) | Unspecified |
|  | Socioeconomic position: general | Cost of resources | Majowicz et al., 2016 (4) | Unspecified |
|  | Socioeconomic position: general | Working conditions | Joffe, 2007 (1) | Positive |
|  | Socioeconomic position: general | Environmental hazards & pollutants | Joffe, 2007 (1); Neff et al., 2009 (22) | Unspecified |
|  | Socioeconomic position: general | Household demographics | Majowicz et al., 2016 (4) | Unspecified |
|  | Socioeconomic position: general | Risk behaviors: general | Ansari et al., 2003 (6) | Unspecified |
|  | Socioeconomic position: general | Healthy diet | Majowicz et al., 2016 (4) | Unspecified |
|  | Socioeconomic position: general | Health-enhancing biological factors | Wittenborn et al., 2015 (3) | Positive |
|  | Socioeconomic position: general | Health-positive psychosocial factors: general | Ansari et al., 2003 (6) | Unspecified |
|  | Socioeconomic position: general | Bandwidth & time | Cavill et al., 2020 (7) | Negative |
|  | Socioeconomic position: general | Health-positive knowledge & skills | Crielaard et al., 2021 (14) | Positive |
|  | Socioeconomic position: general | Social capital | Neff et al., 2009 (22) | Unspecified |
|  | Socioeconomic position: general | Income | Joffe, 2007 (1); Cavill et al., 2020 (7) | Positive |
|  | Socioeconomic position: general | Employment | Brittin et al., 2015 (21); Cavill et al., 2020 (7) | Positive |
|  | Socioeconomic position: general | Education | Joffe, 2007 (1) | Positive |
|  | Socioeconomic position: general | Health-positive cultural and social norms | Ansari et al., 2003 (6); Joffe, 2007 (1); Majowicz et al., 2016 (4) | Unspecified |
|  | Income | General health | Lymer and Brown, 2012 (2);Mahamoud et al., 2013 (8) | Positive |
|  | Income | Mental health | Mooney et al., 2022 (10) | Unspecified |
|  | Income | Stress | Mooney et al., 2022 (10) | Unspecified |
|  | Income | Chronic disease | Brittin et al., 2015 (21);Mahamoud et al., 2013 (8); Mooney et al., 2022 (10); Broomhead et al., 2021 (12) | Negative |
|  | Income | Obesity | Lymer and Brown, 2012 (2) | Unspecified |
|  | Income | Health markers | Chen et al., 2018 (20) | Positive |
|  | Income | Health system attributes: general | Joffe, 2007 (1); Lymer and Brown, 2012 (2) | Unspecified |
|  | Income | Access to healthcare | Mahamoud et al., 2013 (8) | Negative |
|  | Income | Food environment: accessibility | Friel et al., 2017 (15) | Positive |
|  | Income | Physical activity environment: accessibility | Cavill et al., 2020 (7) | Positive |
|  | Income | Built environment | Joffe, 2007 (1) | Unspecified |
|  | Income | Financial strain | Joffe, 2007 (1); Sawyer et al., 2021 (16); Salvo et al., 2022 (28) | Conflicting |
|  | Income | Cost of resources | Reumers et al., 2022 (5) | Negative |
|  | Income | Violence | Mooney et al., 2022 (10) | Unspecified |
|  | Income | Environmental hazards & pollutants | Joffe, 2007 (1) | Unspecified |
|  | Income | Neighborhood income | Orr et al., 2014 (26); Orr et al., 2016 (27) | Positive |
|  | Income | Household demographics | Mooney et al., 2022 (10) | Unspecified |
|  | Income | Housing | Friel et al., 2017 (15); Joffe, 2007 (1); Mahamoud et al., 2013 (8) | Positive |
|  | Income | Risk behaviors: general | Mahamoud et al., 2013 (8) | Negative |
|  | Income | Physical activity | Lymer and Brown, 2012 (2); Yang et al., 2019 (33) | Unspecified |
|  | Income | Alcohol & drug consumption | Holder and Blose, 1987 (25); Lymer and Brown, 2012 (2); Mooney et al., 2022 (10) | Positive |
|  | Income | Smoking | Lymer and Brown, 2012 (2) | Unspecified |
|  | Income | Care-seeking behavior | Lymer and Brown, 2012 (2) | Unspecified |
|  | Income | Other behaviors | Orr et al., 2014 (26); Orr et al., 2016 (27) | Unspecified |
|  | Income | Bandwidth & time | Sawyer et al., 2021 (16) | Positive |
|  | Income | Health-positive tendencies & habits | Blok et al., 2018 (30) | Positive |
|  | Income | Health-positive attitudes, beliefs, and preferences | Auchincloss et al., 2011 (23); Blok et al., 2015 (24) | Positive |
|  | Income | Education | Friel et al., 2017 (15) | Positive |
|  | Income | Generous social policies | Reumers et al., 2022 (5) | Unspecified |
|  | Employment | General health | Joffe, 2007 (1); Lymer and Brown, 2012 (2) | Unspecified |
|  | Employment | Mental health | Occhipinti et al., 2021 (11) | Unspecified |
|  | Employment | Obesity | Lymer and Brown, 2012 (2) | Unspecified |
|  | Employment | Health system attributes: general | Lymer and Brown, 2012 (2) | Unspecified |
|  | Employment | Access to healthcare | Mills et al., 2023 (35) | Positive |
|  | Employment | Treatment | Sturmberg et al., 2017 (18) | Unspecified |
|  | Employment | Financial strain | Mills et al., 2023 (35) | Negative |
|  | Employment | Violence | Occhipinti et al., 2021 (11) | Unspecified |
|  | Employment | Environmental hazards & pollutants | Mills et al., 2023 (35) | Negative |
|  | Employment | Physical activity | Lymer and Brown, 2012 (2); Yang et al., 2019 (33); Cavill et al., 2020 (7) | Positive |
|  | Employment | Alcohol & drug consumption | Lymer and Brown, 2012 (2) | Unspecified |
|  | Employment | Smoking | Lymer and Brown, 2012 (2) | Unspecified |
|  | Employment | Care-seeking behavior | Lymer and Brown, 2012 (2) | Unspecified |
|  | Employment | Bandwidth & time | Friel et al., 2017 (15); Mills et al., 2023 (35); Salvo et al., 2022 (28) | Conflicting |
|  | Employment | Income | Brittin et al., 2015 (21); Reumers et al., 2022 (5) | Positive |
|  | Employment | Employment | Chen et al., 2018 (20) | Conflicting |
|  | Employment | Structural discrimination | Mills et al., 2023 (35) | Negative |
|  | Education | General health | Lymer and Brown, 2012 (2) | Unspecified |
|  | Education | Mental health | Mooney et al., 2022 (10) | Unspecified |
|  | Education | Stress | Mooney et al., 2022 (10) | Unspecified |
|  | Education | Chronic disease | Broomhead et al., 2021 (12) | Unspecified |
|  | Education | Obesity | Lymer and Brown, 2012 (2) | Unspecified |
|  | Education | Health system attributes: general | Lymer and Brown, 2012 (2) | Unspecified |
|  | Education | Treatment | Sturmberg et al., 2017 (18) | Unspecified |
|  | Education | Working conditions | Friel et al., 2017 (15);Joffe, 2007 (1) | Conflicting |
|  | Education | Violence | Mooney et al., 2022 (10) | Unspecified |
|  | Education | Household demographics | Mooney et al., 2022 (10) | Unspecified |
|  | Education | Healthy diet | Orr et al., 2014 (26); Orr et al., 2016 (27); Zhang et al., 2014 (29); Li et al., 2018 (13) | Positive |
|  | Education | Physical activity | Lymer and Brown, 2012 (2); Orr et al., 2016 (27) | Positive |
|  | Education | Alcohol & drug consumption | Lymer and Brown, 2012 (2); Mooney et al., 2022 (10) | Unspecified |
|  | Education | Smoking | Lymer and Brown, 2012 (2) | Unspecified |
|  | Education | Care-seeking behavior | Lymer and Brown, 2012 (2) | Unspecified |
|  | Education | Other behaviors | Joffe, 2007 (1) | Unspecified |
|  | Education | Bandwidth & time | Friel et al., 2017 (15) | Positive |
|  | Education | Health-positive attitudes, beliefs, and preferences | Li et al., 2018 (13) | Positive |
|  | Education | Social capital | Friel et al., 2017 (15) | Positive |
|  | Education | Income | Orr et al., 2014 (26); Orr et al., 2016 (27); Mooney et al., 2022 (10) | Positive |
|  | Education | Employment | Reumers et al., 2022 (5) | Positive |
|  | Education | Education | Joffe, 2007 (1) | Positive |
|  | Disadvantage | General health | Milstein et al., 2010 (19) | Negative |
|  | Disadvantage | Short-term & infectious disease | Milstein et al., 2010 (19) | Positive |
|  | Disadvantage | Access to healthcare | Milstein et al., 2010 (19) | Negative |
|  | Disadvantage | Environmental hazards & pollutants | Milstein et al., 2010 (19) | Positive |
|  | Disadvantage | Risk behaviors: general | Milstein et al., 2010 (19) | Positive |
|  | Sex & gender: female | General health | Yang et al., 2019 (33) | Unspecified |
|  | Sex & gender: female | Mental health | Mooney et al., 2022 (10) | Unspecified |
|  | Sex & gender: female | Stress | Mooney et al., 2022 (10) | Unspecified |
|  | Sex & gender: female | Obesity | Majowicz et al., 2016 (4) | Unspecified |
|  | Sex & gender: female | Health system attributes: general | Lymer and Brown, 2012 (2) | Unspecified |
|  | Sex & gender: female | Healthcare costs | Lymer and Brown, 2012 (2) | Unspecified |
|  | Sex & gender: female | Financial strain | Majowicz et al., 2016 (4) | Unspecified |
|  | Sex & gender: female | Violence | Mooney et al., 2022 (10) | Unspecified |
|  | Sex & gender: female | Household demographics | Mooney et al., 2022 (10) | Unspecified |
|  | Sex & gender: female | Healthy diet | Majowicz et al., 2016 (4); Zhang et al., 2014 (29); Li et al., 2018 (13) | Positive |
|  | Sex & gender: female | Care-seeking behavior | Lymer and Brown, 2012 (2) | Unspecified |
|  | Sex & gender: female | Health-enhancing biological factors | Majowicz et al., 2016 (4) | Unspecified |
|  | Sex & gender: female | Health-positive tendencies & habits | Blok et al., 2018 (30) | Unspecified |
|  | Sex & gender: female | Health-positive attitudes, beliefs, and preferences | Li et al., 2018 (13) | Conflicting |
|  | Sex & gender: female | Social capital | Majowicz et al., 2016 (4) | Unspecified |
|  | Sex & gender: female | Income | Mooney et al., 2022 (10) | Unspecified |
|  | Sex & gender: female | Employment | Yang et al., 2019 (33) | Unspecified |
|  | Ethnicity | Mental health | Mooney et al., 2022 (10) | Unspecified |
|  | Ethnicity | Stress | Mooney et al., 2022 (10) | Unspecified |
|  | Ethnicity | Obesity | Majowicz et al., 2016 (4) | Unspecified |
|  | Ethnicity | Financial strain | Majowicz et al., 2016 (4) | Unspecified |
|  | Ethnicity | Violence | Mooney et al., 2022 (10) | Unspecified |
|  | Ethnicity | Household demographics | Mooney et al., 2022 (10) | Unspecified |
|  | Ethnicity | Healthy diet | Majowicz et al., 2016 (4) | Unspecified |
|  | Ethnicity | Alcohol & drug consumption | Mooney et al., 2022 (10) | Unspecified |
|  | Ethnicity | Social capital | Majowicz et al., 2016 (4) | Unspecified |
|  | Ethnicity | Income | Mooney et al., 2022 (10) | Unspecified |
| Structural factors | Regulatory governance | Obesity | Majowicz et al., 2016 (4) | Unspecified |
|  | Regulatory governance | Food environment: general | Majowicz et al., 2016 (4) | Unspecified |
|  | Regulatory governance | Food environment: availability | Majowicz et al., 2016 (4) | Unspecified |
|  | Regulatory governance | Food environment: accessibility | Friel et al., 2017 (15); Weiler et al., 2015 (9) | Positive |
|  | Regulatory governance | Food environment: marketing & advertising | Friel et al., 2017 (15); Majowicz et al., 2016 (4) | Positive |
|  | Regulatory governance | Built environment | Friel et al., 2017 (15); Majowicz et al., 2016 (4) | Positive |
|  | Regulatory governance | Financial strain | Majowicz et al., 2016 (4) | Unspecified |
|  | Regulatory governance | Cost of resources | Majowicz et al., 2016 (4) | Unspecified |
|  | Regulatory governance | Working conditions | Friel et al., 2017 (15) | Positive |
|  | Regulatory governance | Environmental hazards & pollutants | Majowicz et al., 2016 (4) | Unspecified |
|  | Regulatory governance | Household demographics | Majowicz et al., 2016 (4) | Unspecified |
|  | Regulatory governance | Health-positive knowledge & skills | Majowicz et al., 2016 (4) | Unspecified |
|  | Regulatory governance | Social cohesion | Majowicz et al., 2016 (4) | Unspecified |
|  | Regulatory governance | Market-focused economic environment | Friel et al., 2017 (15) | Negative |
|  | Regulatory governance | Health-positive cultural and social norms | Majowicz et al., 2016 (4) | Unspecified |
|  | Neo-liberal macroeconomic policies | Obesity | Majowicz et al., 2016 (4) | Unspecified |
|  | Neo-liberal macroeconomic policies | Food environment: availability | Friel et al., 2017 (15) | Negative |
|  | Neo-liberal macroeconomic policies | Built environment | Majowicz et al., 2016 (4) | Unspecified |
|  | Neo-liberal macroeconomic policies | Financial strain | Majowicz et al., 2016 (4) | Unspecified |
|  | Neo-liberal macroeconomic policies | Healthy diet | Majowicz et al., 2016 (4) | Unspecified |
|  | Neo-liberal macroeconomic policies | Physical activity | Majowicz et al., 2016 (4) | Unspecified |
|  | Neo-liberal macroeconomic policies | Market-focused economic environment | Majowicz et al., 2016 (4) | Unspecified |
|  | Neo-liberal macroeconomic policies | Health-positive cultural and social norms | Majowicz et al., 2016 (4) | Unspecified |
|  | Generous social policies | Cost of resources | Friel et al., 2017 (15); Reumers et al., 2022 (5) | Negative |
|  | Generous social policies | Care-seeking behavior | Reumers et al., 2022 (5) | Positive |
|  | Generous social policies | Income | Friel et al., 2017 (15); Reumers et al., 2022 (5) | Positive |
|  | Generous social policies | Generous social policies | Friel et al., 2017 (15) | Positive |
|  | Generous social policies | Citizen engagement | Reumers et al., 2022 (5) | Positive |
|  | Restrictive public policies | General physical environment: availability | Mills et al., 2023 (35) | Positive |
|  | Restrictive public policies | Alcohol & drug consumption | Holder and Blose, 1987 (25) | Negative |
|  | Market-focused economic environment | Obesity | Majowicz et al., 2016 (4) | Unspecified |
|  | Market-focused economic environment | Food environment: availability | Friel et al., 2017 (15); Majowicz et al., 2016 (4); Sawyer et al., 2021 (16) | Conflicting |
|  | Market-focused economic environment | Food environment: accessibility | Friel et al., 2017 (15) | Negative |
|  | Market-focused economic environment | Food environment: marketing & advertising | Friel et al., 2017 (15) | Negative |
|  | Market-focused economic environment | General physical environment: availability | Mills et al., 2023 (35) | Negative |
|  | Market-focused economic environment | General physical environment: marketing & advertising | Friel et al., 2017 (15); Mills et al., 2023 (35) | Negative |
|  | Market-focused economic environment | Built environment | Majowicz et al., 2016 (4) | Unspecified |
|  | Market-focused economic environment | Financial strain | Majowicz et al., 2016 (4) | Unspecified |
|  | Market-focused economic environment | Cost of resources | Friel et al., 2017 (15); Reumers et al., 2022 (5) | Conflicting |
|  | Market-focused economic environment | Environmental hazards & pollutants | Mills et al., 2023 (35) | Positive |
|  | Market-focused economic environment | Household demographics | Majowicz et al., 2016 (4) | Unspecified |
|  | Market-focused economic environment | Health-positive tendencies & habits | Mills et al., 2023 (35) | Negative |
|  | Market-focused economic environment | Health-positive attitudes, beliefs, and preferences | Mills et al., 2023 (35) | Negative |
|  | Market-focused economic environment | Health-positive cultural and social norms | Majowicz et al., 2016 (4); Mills et al., 2023 (35) | Negative |
|  | Health-positive cultural and social norms | General health | Ansari et al., 2003 (6); Joffe, 2007 (1); Majowicz et al., 2016 (4); Weiler et al., 2015 (9) | Unspecified |
|  | Health-positive cultural and social norms | Obesity | Majowicz et al., 2016 (4) | Unspecified |
|  | Health-positive cultural and social norms | Health system attributes: general | Ansari et al., 2003 (6) | Unspecified |
|  | Health-positive cultural and social norms | Food environment: availability | Friel et al., 2017 (15) | Positive |
|  | Health-positive cultural and social norms | Food environment: accessibility | Weiler et al., 2015 (9) | Unspecified |
|  | Health-positive cultural and social norms | Built environment | Majowicz et al., 2016 (4); Cavill et al., 2020 (7) | Positive |
|  | Health-positive cultural and social norms | Financial strain | Majowicz et al., 2016 (4) | Unspecified |
|  | Health-positive cultural and social norms | Environmental hazards & pollutants | Cavill et al., 2020 (7) | Negative |
|  | Health-positive cultural and social norms | Household demographics | Majowicz et al., 2016 (4) | Unspecified |
|  | Health-positive cultural and social norms | Risk behaviors: general | Ansari et al., 2003 (6) | Unspecified |
|  | Health-positive cultural and social norms | Healthy diet | Friel et al., 2017 (15); Majowicz et al., 2016 (4) | Positive |
|  | Health-positive cultural and social norms | Physical activity | Cavill et al., 2020 (7) | Positive |
|  | Health-positive cultural and social norms | Alcohol & drug consumption | Holder and Blose, 1987 (25) | Unspecified |
|  | Health-positive cultural and social norms | Smoking | Mills et al., 2023 (35) | Negative |
|  | Health-positive cultural and social norms | Health-positive psychosocial factors: general | Ansari et al., 2003 (6) | Unspecified |
|  | Health-positive cultural and social norms | Health-positive tendencies & habits | Friel et al., 2017 (15) | Positive |
|  | Health-positive cultural and social norms | Health-positive attitudes, beliefs, and preferences | Friel et al., 2017 (15) | Positive |
|  | Health-positive cultural and social norms | Social capital | Mills et al., 2023 (35) | Positive |
|  | Health-positive cultural and social norms | Socioeconomic position: general | Ansari et al., 2003 (6) | Unspecified |
|  | Health-positive cultural and social norms | Regulatory governance | Majowicz et al., 2016 (4); Weiler et al., 2015 (9) | Unspecified |
|  | Health-positive cultural and social norms | Health-positive cultural and social norms | Majowicz et al., 2016 (4);D2293; Cavill et al., 2020 (7) | Conflicting |
|  | Health-positive cultural and social norms | Structural discrimination | Mills et al., 2023 (35) | Negative |
|  | Stigma | Health-positive attitudes, beliefs, and preferences | Reumers et al., 2022 (5) | Negative |
|  | Stigma | Social capital | Sawyer et al., 2021 (16) | Negative |
|  | Structural discrimination | Stress | Mills et al., 2023 (35) | Positive |
|  | Structural discrimination | Access to healthcare | Mills et al., 2023 (35) | Negative |
|  | Structural discrimination | General physical environment: availability | Mills et al., 2023 (35) | Negative |
|  | Structural discrimination | General physical environment: marketing & advertising | Mills et al., 2023 (35) | Negative |
|  | Structural discrimination | Financial strain | Mills et al., 2023 (35) | Positive |
|  | Structural discrimination | Housing | Mills et al., 2023 (35) | Negative |
|  | Structural discrimination | Bandwidth & time | Mills et al., 2023 (35) | Negative |
|  | Structural discrimination | Social capital | Mills et al., 2023 (35) | Negative |
|  | Structural discrimination | Employment | Mills et al., 2023 (35) | Negative |
|  | Structural discrimination | Restrictive public policies | Mills et al., 2023 (35) | Positive |
|  | Structural discrimination | Structural discrimination | Mills et al., 2023 (35) | Positive |
|  | Citizen engagement | Food environment: accessibility | Friel et al., 2017 (15) | Negative |
|  | Citizen engagement | Learning environment | Friel et al., 2017 (15) | Positive |
|  | Citizen engagement | Social capital | Reumers et al., 2022 (5) | Positive |
|  | Citizen engagement | Regulatory governance | Friel et al., 2017 (15) | Positive |
|  | Citizen engagement | Citizen engagement | Friel et al., 2017 (15) | Positive |

# References

1. Joffe M. Health, Livelihoods, and Nutrition in Low-Income Rural Systems. Food Nutr Bull. 2007;28(2_suppl2):S227–36.

2. Lymer S, Brown L. Developing a Dynamic Microsimulation Model of the Australian Health System: A Means to Explore Impacts of Obesity over the Next 50 Years. Epidemiology Research International. 2012;2012:1–13.

3. Wittenborn AK, Rahmandad H, Rick J, Hosseinichimeh N. Depression as a systemic syndrome: mapping the feedback loops of major depressive disorder. Psychol Med. 2016;46(3):551–62.

4. Majowicz SE, Meyer SB, Kirkpatrick SI, Graham JL, Shaikh A, Elliott SJ, et al. Food, health, and complexity: towards a conceptual understanding to guide collaborative public health action. BMC Public Health. 2016;16(1):487.

5. Reumers L, Bekker M, Hilderink H, Jansen M, Helderman JK, Ruwaard D. Qualitative modelling of social determinants of health using group model building: the case of debt, poverty, and health. Int J Equity Health. 2022 Dec;21(1):72.

6. Ansari Z, Carson NJ, Ackland MJ, Vaughan L, Serraglio A. A public health model of the social determinants of health. Social and Preventive Medicine. 2003;48(4):242–51.

7. Cavill N, Richardson D, Faghy M, Bussell C, Rutter H. Using System Mapping to Help Plan and Implement City-Wide Action to Promote Physical Activity. Journal of Public Health Research. 2020;9(3):jphr.2020.1759.

8. Mahamoud A, Roche B, Homer J. Modelling the social determinants of health and simulating short-term and long-term intervention impacts for the city of Toronto, Canada. Social Science & Medicine. 2013;93:247–55.

9. Weiler AM, Hergesheimer C, Brisbois B, Wittman H, Yassi A, Spiegel JM. Food sovereignty, food security and health equity: a meta-narrative mapping exercise. Health Policy Plan. 2015;30(8):1078–92.

10. Mooney SJ, Shev AB, Keyes KM, Tracy M, Cerdá M. G-Computation and Agent-Based Modeling for Social Epidemiology: Can Population Interventions Prevent Posttraumatic Stress Disorder? American Journal of Epidemiology. 2022;191(1):188–97.

11. Occhipinti JA, Skinner A, Iorfino F, Lawson K, Sturgess J, Burgess W, et al. Reducing youth suicide: systems modelling and simulation to guide targeted investments across the determinants. BMC Med. 2021;19(1):61.

12. Broomhead T, Ballas D, Baker SR. Neighbourhoods and oral health: Agent-based modelling of tooth decay. Health & Place. 2021;71:102657.

13. Li Y, Zhang D, Thapa JR, Madondo K, Yi S, Fisher E, et al. Assessing the role of access and price on the consumption of fruits and vegetables across New York City using agent-based modeling. Preventive Medicine. 2018;106:73–8.

14. Crielaard L, Nicolaou M, Sawyer A, Quax R, Stronks K. Understanding the impact of exposure to adverse socioeconomic conditions on chronic stress from a complexity science perspective. BMC Med. 2021;19(1):242.

15. Friel S, Pescud M, Malbon E, Lee A, Carter R, Greenfield J, et al. Using systems science to understand the determinants of inequities in healthy eating. PLoS ONE. 2017;12(11).

16. Sawyer ADM, Van Lenthe F, Kamphuis CBM, Terragni L, Roos G, Poelman MP, et al. Dynamics of the complex food environment underlying dietary intake in low-income groups: a systems map of associations extracted from a systematic umbrella literature review. Int J Behav Nutr Phys Act. 2021 Dec;18(1):96.

17. Homa L, Rose J, Hovmand PS, Cherng ST, Riolo RL, Kraus A, et al. A Participatory Model of the Paradox of Primary Care. The Annals of Family Medicine. 2015;13(5):456–65.

18. Sturmberg JP, Bennett JM, Martin CM, Picard M. ‘Multimorbidity’ as the manifestation of network disturbances: ‘Multimorbidity’ as network disturbances. J Eval Clin Pract. 2017;23(1):199–208.

19. Milstein B, Homer J, Hirsch G. Analyzing National Health Reform Strategies With a Dynamic Simulation Model. Am J Public Health. 2010;100(5):811–9.

20. Chen HJ, Xue H, Liu S, Huang TTK, Wang YC, Wang Y. Obesity trend in the United States and economic intervention options to change it: A simulation study linking ecological epidemiology and system dynamics modeling. Public Health. 2018;161:20–8.

21. Brittin J, Araz OM, Nam Y, Huang TK. A system dynamics model to simulate sustainable interventions on chronic disease outcomes in an urban community. Journal of Simulation. 2015;9(2):140–55.

22. Neff RA, Palmer AM, McKenzie SE, Lawrence RS. Food Systems and Public Health Disparities. Journal of Hunger & Environmental Nutrition. 2009;4(3–4):282–314.

23. Auchincloss A, Riolo RL, Brown DG, Cook J, Diez Roux AV. An agent-based model of income inequalities in diet in the context of residential segregation. American Journal of Preventive Medicine. 2011;40(3):303–11.

24. Blok DJ, De Vlas SJ, Bakker R, Van Lenthe FJ. Reducing Income Inequalities in Food Consumption. American Journal of Preventive Medicine. 2015;49(4):605–13.

25. Holder HD, Blose JO. Reduction of community alcohol problems: computer simulation experiments in three counties. J Stud Alcohol. 1987;48(2):124–35.

26. Orr MG, Galea S, Riddle M, Kaplan GA. Reducing racial disparities in obesity: simulating the effects of improved education and social network influence on diet behavior. Annals of Epidemiology. 2014;24(8):563–9.

27. Orr MG, Kaplan GA, Galea S. Neighbourhood food, physical activity, and educational environments and black/white disparities in obesity: a complex systems simulation analysis. J Epidemiol Community Health. 2016;70(9):862–7.

28. Salvo D, Lemoine P, Janda KM, Ranjit N, Nielsen A, Van Den Berg A. Exploring the Impact of Policies to Improve Geographic and Economic Access to Vegetables among Low-Income, Predominantly Latino Urban Residents: An Agent-Based Model. Nutrients. 2022;14(3):646.

29. Zhang D, Giabbanelli PJ, Arah OA, Zimmerman FJ. Impact of Different Policies on Unhealthy Dietary Behaviors in an Urban Adult Population: An Agent-Based Simulation Model. Am J Public Health. 2014;104(7):1217–22.

30. Blok DJ, Van Lenthe FJ, De Vlas SJ. The impact of individual and environmental interventions on income inequalities in sports participation: explorations with an agent-based model. Int J Behav Nutr Phys Act. 2018;15(1):107.

31. Yang Y, Auchincloss AH, Rodriguez DA, Brown DG, Riolo R, Diez-Roux AV. Modeling spatial segregation and travel cost influences on utilitarian walking: Towards policy intervention. Computers, Environment and Urban Systems. 2015;51:59–69.

32. Yang Y, Diez Roux AV, Auchincloss AH, Rodriguez DA, Brown DG. A Spatial Agent-Based Model for the Simulation of Adults’ Daily Walking Within a City. American Journal of Preventive Medicine. 2011;40(3):353–61.

33. Yang Y, Langellier BA, Stankov I, Purtle J, Nelson KL, Diez Roux AV. Examining the possible impact of daily transport on depression among older adults using an agent-based model. Aging & Mental Health. 2019;23(6):743–51.

34. Rahmani J, Mirzay Razaz J, Kalantari N, Garcia LMT, Shariatpanahi SP, Bawadi H, et al. Dynamic conceptual framework to investigate adoption of healthy diet through agent-based modelling. BFJ. 2021;123(8):2743–55.

35. Mills SD, Golden SD, O’Leary MC, Logan P, Hassmiller Lich K. Using systems science to advance health equity in tobacco control: a causal loop diagram of smoking. Tob Control. 2023;32(3):287–95.
